# Non-destructive estimation of maize carotenoids using reflectance-based spectral indices

**DOI:** 10.3389/fpls.2026.1699049

**Published:** 2026-02-12

**Authors:** Attila Nagy, Ahmed Elbeltagi, László Radócz, János Tamás, Andrea Szabó

**Affiliations:** 1 Faculty of Agricultural and Food Sciences and Environmental Management, Institute of Water and Environmental Management, University of Debrecen, Debrecen, Hungary; 2 National Laboratory for Water Science and Water Safety, Faculty of Agricultural and Food Sciences and Environmental Management, Institute of Water and Environmental Management, University of Debrecen, Debrecen, Hungary; 3 Agricultural Engineering Dept., Faculty of Agriculture, Mansoura University, Mansoura, Egypt

**Keywords:** hyperspectral indices, maize carotenoid estimation, MRMR algorithm, pigment content, spectral bands

## Abstract

This study investigates the relationship between maize leaf carotenoid content and spectral reflectance, evaluates existing carotenoid estimation indices, and develops new spectral indices and machine learning models for improved prediction. A strong positive correlation was observed between carotenoid and chlorophyll content, highlighting carotenoids’ role in both light harvesting and photoprotection. Spectral analysis revealed that carotenoid concentration significantly affects leaf reflectance in the visible range, particularly between 500–650 nm. Existing carotenoid indices exhibited limited predictive performance for the studied samples, prompting the development of nine new indices based on principal component analysis. Among these, CAR_7_, CAR_8_, and CAR_9_ demonstrated superior predictive ability across different training (2021-2022: R^2^ = 0.72-0.76, NRMSE = 15-16%, 2021-2023: R^2^ = 0.60-0.62, NRMSE = 11-12%, 2022-2023: R^2^ = 0.42-0.49, NRMSE = 18.3-18.5%) and testing periods (2023: R^2^ = 0.44-0.50, NRMSE = 14-19%, 2022: R^2^ = 0.65-0.72, NRMSE = 13-16%, 2021: R^2^ = 0.81-0.83, NRMSE = 18.28-24.65%). Machine learning models further improved carotenoid estimation, with REPTree providing the most reliable and balanced performance during testing (R^2^ = 0.79, NRMSE = 13.84%). The findings suggest that the combination of targeted spectral indices and appropriate machine learning approaches enables accurate, non-destructive estimation of maize carotenoid content, offering potential for practical applications in crop monitoring and stress assessment.

## Graphical Abstract

**Figure:**
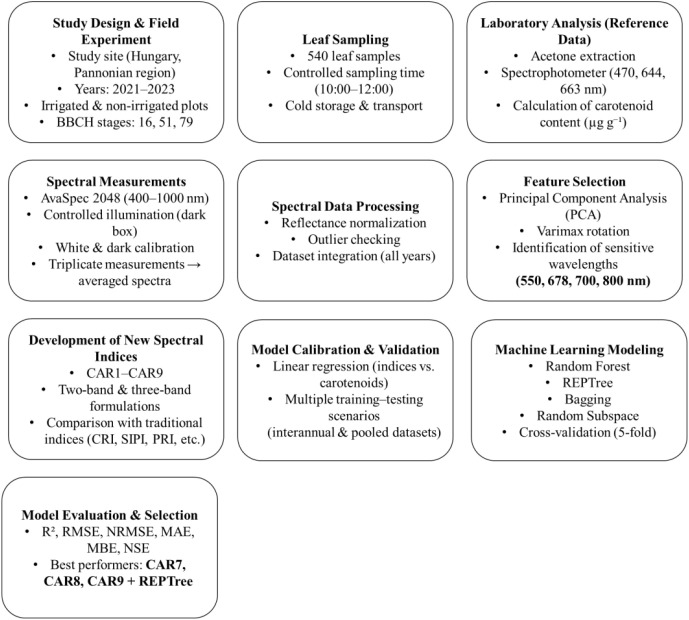
The abstract should ideally be structured according to the IMRaD format (Introduction, Methods, Results and Discussion). Provide a structured abstract if possible. If your article has been copyedited by us, please provide the updated abstract based on this version.

## 1 Introduction

Globalization and digitalization are having an increasing impact on food production and agricultural practices (Akram et al., 2021). In this context, precision agriculture is an increasingly widely used technology that enables the optimization of production resources, increased yields, and reduced environmental impact of agricultural activities (Sishodia et al., 2020; Monteiro et al., 2021; McFadden et al., 2023; Getahun et al., 2024; Nath, 2024). The ever-growing global population and the associated demand for food are driving the need to increase the efficiency of food production, making all agricultural activities—including the cultivation of corn (Zea mays) —of paramount importance in terms of food security (Cox, 2002; Fountas et al., 2015; Erenstein et al., 2022). Corn is particularly important in Europe for food supply and animal feed, and it is one of the most widely grown crops in Hungary (Erenstein et al., 2022). Accurate, non-destructive monitoring of the biological processes and pigments of this plant is key to precision farming practices. Among the pigments involved in photosynthesis, carotenoids play a key role in light absorption and protection against oxidative damage caused by reactive oxygen species (Hashimoto et al., 2016; Sies et al., 2022). These pigments adapt dynamically to light conditions: lutein dominates in the shade, while β-carotene and xanthophyll cycle pigments increase under high light conditions (Matsubara et al., 2009). Carotenoid content and the carotenoid-chlorophyll ratio can remain stable despite changes in pigments (Hallik et al., 2012), and shifts in the chlorophyll a-b ratio may indicate changes in light quality and quantity. Spectrally, carotenoids such as β-carotene absorb in the blue (450–485 nm) and green (500–565 nm) wavelength ranges (Mikhailenko, 2022). The spectral characteristics of pigments can be accurately measured using remote sensing and spectroscopy (Berardo et al., 2004), which allow the monitoring of carotenoid concentrations among different corn varieties, as well as the monitoring of plant nutrient and water status and the detection of early diseases (Zahir et al., 2022). Spectral techniques such as VIS–NIR measurements provide a cost-effective, non-destructive method for real-time monitoring of pigment content (Davey et al., 2009; Žilić et al., 2012). Several spectral indices already exist for estimating the carotenoid content of corn, such as CRI550/CRI700 (Gitelson et al., 2002), CARRedEdge, CARGreen (Gitelson et al., 2006), CARI (Zhou et al., 2017), PRI (Gamon et al., 1992), PSRI (Merzlyak et al., 1999), and CCRI (Zhou et al., 2019). These indices improve nutrient analysis, stress detection, and crop quality prediction (Sharma et al., 2020). The application of machine learning allows spectral data to be more accurately linked to carotenoid concentration (Dong and Wang, 2024). The combination of hyperspectral data and machine learning models offers significant advantages over traditional methods (Ang and Seng, 2021; Zhang et al., 2021; Sun et al., 2022), as the analysis of high-dimensional spectral data allows for the detection of subtle spectral differences related to carotenoid levels and plant health (Gao et al., 2020).

Previous research has mainly relied on general spectral indices and reference models that do not take into account the specific morphological and physiological characteristics of corn. Furthermore, the use of simpler models is not always justified and often leads to overfitting (Aghighi et al., 2018; Gao et al., 2018; Ramos et al., 2020; Zhan et al., 2024), while PLSR, as the gold standard, is rarely included in combined analyses. On the otherhand Principal Component Analysis (PCA) and machine learning models estimate accurately maize parameters correlating with canopy carotene content from spectral data (Da Silva et al., 2024). Random Forest is a highly accurate and robust method for handling large datasets. Ramos et al, 2020 (Gao et al., 2018) used it for maize yield prediction and combined RF techniques with UAV-based spectral data. However, Random Forest is also significant in terms of plant protection. Gao J. et al., 2018 (Zhan et al., 2024) used it for weed species assessment, applying the RF technique in combination with NIR hyperspectral data. The Bagging ML technique is often used to improve the models’ performance, or also for maize yield prediction (Aghighi et al., 2018; Da Silva et al., 2024). Da Silva et al., 2024 (Baio et al., 2022) used REPtree combined with hyperspectral datasets to estimate different pigment ratios in maize, including carotenoid content. Also, they were interested in making a new approach for a predictive nitrogen supply model. However, REPtree has also been applied in several scientific studies, for example, in maize yield prediction/estimation, as well as in optimizing irrigation, water application, and improving water management efficiency (Yang et al., 2023; Killeen et al., 2024). The Random Subspace (RS) ML technique, which has been primarily used in maize-related research for distinguishing maize hybrids, and for plant protection purposes, RS was used for identifying different weed species from hyperspectral datasets (Hungarian National Weather Service (HNWS), 2020). Thus, there is a lack of a testable hypothesis linking the development of corn-specific spectral indices and combining them with ML techniques with improved accuracy in carotenoid estimation. Therefore the hypothesisof this study to optimize corn-specific spectral indices improves the accuracy of non-destructive carotenoid content estimation compared to traditional, general indices.

The aim of this study is to develop and validate new corn-specific spectral indices and to apply several machine learning models, including Random Forest, Bagging, REPtree, and Random Subspace, to predict the relationship between the indices and carotenoid concentration (Aghighi et al., 2018; Gao et al., 2018; Hungarian National Weather Service (HNWS), 2020; Baio et al., 2022; Yang et al., 2023; Da Silva et al., 2024; Killeen et al., 2024; Zhan et al., 2024). We compare the performance of these models based on statistical indicators such as R², RMSE, NRMSE, MBE, MAE, and NSE in order to select the most reliable prediction system.

## 2 Materials and methods

### 2.1 Study site

**Figure 1:**
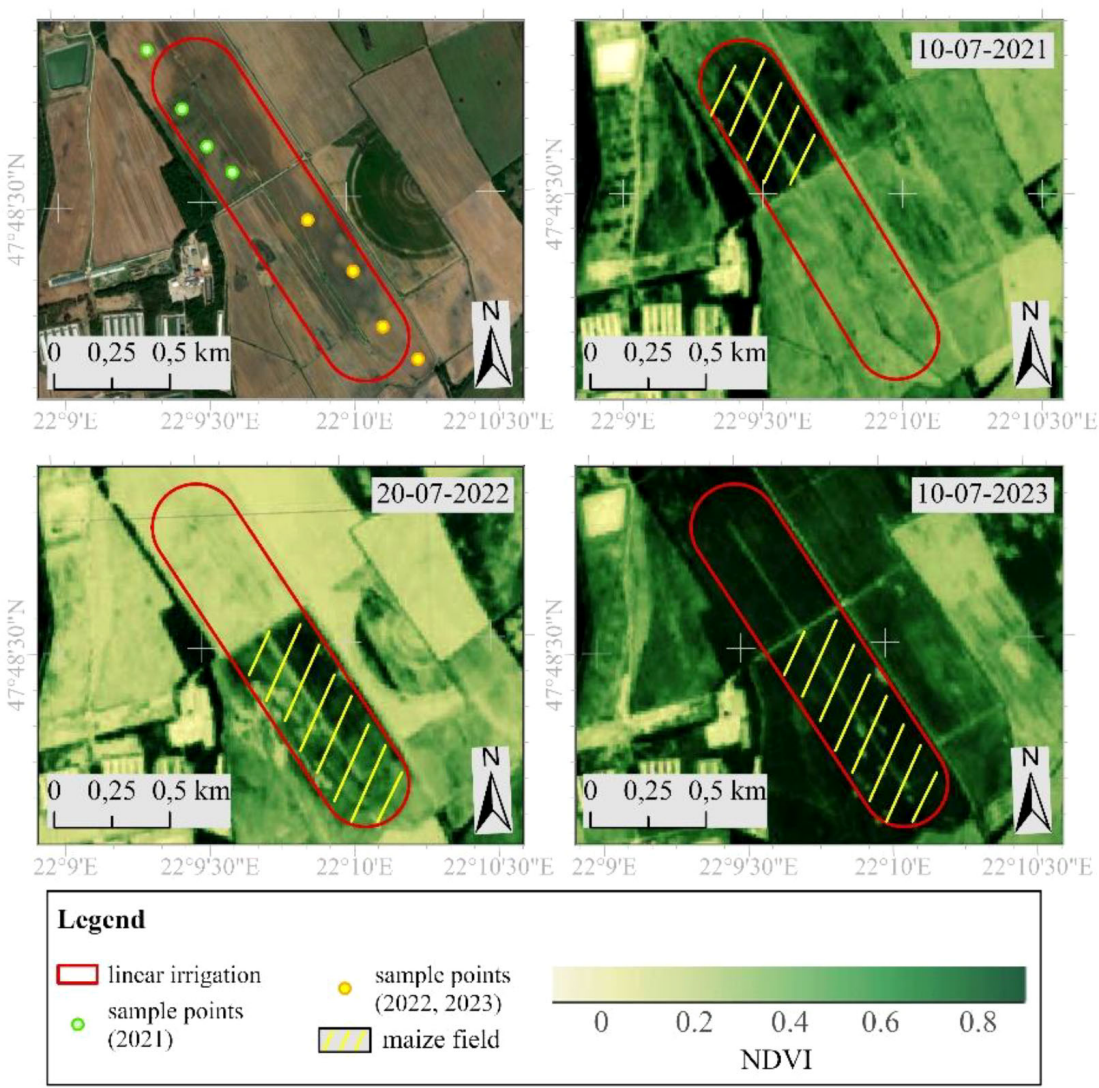
Study site.

The study site is located in the Pannonian region on the Northern Great Plain in Hungary (47°48’18.60”N latitude and 22°9’43.89”E longitude) with an altitude of 144 meters. It lies at the transition of a moderately warm and cold (continental) climate zone. The terrain consists of an alluvial cone plain predominantly covered with sand. Spanning 87.5 hectares, the area is designated as irrigated arable land and features a linear irrigation system (**Figure 1**). Due to past melioration and drainage activities in the previous century, the active water network is now sparse, and the landscape exhibits minimal horizontal fragmentation. In 2010, the European Commission classified the site as a nitrate-vulnerable area. Over the past decade, annual sunshine hours have ranged between 1.900 and 2.000, with around 800 hours in summer and 170 in winter, based on data from the Hungarian National Weather Service (Nagy et al., 2024). The average annual temperature varies between 9.6 °C and 12.6 °C, with summer highs exceeding 34 °C and winter lows dropping below -17.0 °C. Annual precipitation measures between 570 and 600 mm, with approximately 350–360 mm falling during the summer months. The predominant wind directions are from the northeast and southeast, with an average speed of 2.5 m/s (Magyar et al., 2023). In 2021, normal weather conditions were adequate in the area under study, while 2022 saw an extremely severe drought, and 2023 was a much wetter year. During the years the P0725 (FAO 580) corn variety was sown in the area. In 2021, it was sown on May 13, with a sowing density of 76, 000 seeds/hectare and a row spacing of 76.2 cm. Harvesting took place on September 22–23, with an average yield of 34.14 t/ha and a dry matter content of approximately 35%. In 2022, corn was sown on April 19, with a sowing density of 76, 000 seeds/hectare and a row spacing of 75 cm. Harvesting took place on August 23, with an average yield of 34.40 t/ha. In 2023, it was sown on April 29 with a sowing density of 72, 000 seeds/hectare and a row spacing of 75 cm, and harvested on August 16. The silage yield was 40.4 t/ha in irrigated areas and 32.8 t/ha in non-irrigated areas. The corn was grown on sandy soil with extreme water conditions, which made the plant very sensitive to water shortage and heat stress (Szabó et al., 2021).

### 2.2 Measurement method and data processing

Sampling was conducted nine times (three times a year based on BBCH 16, 51, 79) in 2021, 2022, and 2023 across five designated areas, resulting in a total of 540 samples collected from both irrigated and non-irrigated zones. One sampling area was located in the non-irrigated section of the field, while the remaining four were in irrigated sections, selected based on varying soil physical properties (Lichtenthaler and Wellbum, 1983). Sampling took place between 10:00 and 12:00, with 12 samples collected from each designated point. Leaf samples were analyzed in the laboratory within six hours of collection, following storage and transport at 4 °C. For homogenization, samples were processed using 80% acetone and 1 g of quartz sand. After extraction, the suspensions were centrifuged at 3.000 rpm for three minutes, and the clear solution was transferred to a 2.5 ml cuvette. The absorbance of the solution was then measured using a spectrophotometer (SECOMAN Anthelie Light II) at wavelengths of 470 nm, 644 nm, and 663 nm (Szabó et al., 2019).

The carotenoid values were calculated based on the following equation (Szabó et al., 2019):


$$\begin{array}{c} Carotenoid\;\mu g/g\;fresh\;weight=(1000*A_{470nm}–3.27\;\left(12.21*A_{663nm}–2.81*A_{644nm}\right)\\ –104*\left(20.13A_{644nm}–5.03A_{663nm}\right))/229/\left(V/w\right) \end{array}$$

where:

V = volume of tissue extract (ml).

w = fresh weight of tissue (g).

A = absorbance.

### 2.3 Spectral analysis

The AvaSpec 2048 spectrometer was used to record spectral data from leaf samples within the 400–1000 nm wavelength range, with a precision of 0.6 nm. This range was selected for measuring carotenoid levels in maize orchards as it effectively captures the carotenoid absorption maxima and the near-infrared (NIR) region, where plant tissues exhibit high reflectance. Broader wavelength ranges include regions that are irrelevant for carotenoid assessment, such as the ultraviolet (300–400 nm) and mid-infrared (above 1000 nm), which do not provide useful carotenoid data and may introduce measurement noise. The setup consisted of the spectrometer, an AvaLight-HAL halogen light source, and a patented sampling box designed to ensure complete darkness during measurements, preventing interference from external light. Since ambient light sources, such as LEDs or fluorescent bulbs, can affect reflectance at specific wavelengths, eliminating these background influences was crucial for accuracy. To further enhance the reliability of measurements, the spectrometer was calibrated using both white and dark references before data collection, ensuring that recorded reflectance values were normalized against a known reflectance baseline. The dark reference was measured in the absence of light to correct for electronic noise within the spectrometer. Each leaf sample was illuminated and measured in triplicate to account for potential variability in spectral readings due to leaf surface texture, pigmentation heterogeneity, and minor fluctuations in the light source. The averaged spectra from these repeated measurements provided a more robust estimation of carotenoid content. Additionally, leaf samples were kept at a consistent orientation during scanning to minimize the impact of leaf structure on reflectance variability (Liu et al., 2019).

### 2.4 Model building and performance assessment

The results were analyzed statistically using SPSS software, applying PCA with Varimax rotation to condense the data, identify outliers, and uncover patterns and internal structures within the dataset. This approach aimed to pinpoint the wavelengths with the greatest variation in factor weights. Given that the primary changes in leaf samples were related to pigments, particularly carotenoids, variations in reflectance were likely influenced by differences in chlorophyll content. As a result, PCA was well-suited for identifying wavelengths sensitive to plant carotenoid levels (Jolliffe, 2002). Varimax rotation, which optimizes factor loadings by distributing them separately, enabled the clear assignment of individual objects to specific factors (Penuelas and Baret, 1995). As an orthogonal rotation method, Varimax was chosen to enhance the interpretability of PCA results by maximizing the variance of squared loadings for each component. This produced clearer and more distinct patterns, facilitating the identification and analysis of key features within the hyperspectral data. Ultimately, this approach improved both the clarity and reliability of the analysis in this context. The PCA input parameters consist of 1063 variables, including reflectance percentage values from leaf samples across the 400–1000 nm spectrum. Wavelengths were chosen based on the factor weight results of this component, focusing on those with the highest factor weight to identify the most sensitive spectral regions. Furthermore, when selecting wavelengths with low pigment sensitivity, it is important to consider backscattering effects and non-pigment-related interactions (Blackburn, 2007). In the research, PCA is not part of the prediction model, but rather a preliminary scientific variable selection method that searches for spectral characteristics – this is essentially like a feature importance analysis. Then, proprietary vegetation indices were established from the data obtained, which does not transfer the test data information into the model.

Carotenoid estimator models were developed using wavelengths with high and low sensitivity to carotenoids. Both two-band (Gamon et al., 1990) and three-band (Smith, 2020) models capture pigment absorption patterns in narrow spectral regions. Two-band indices (e.g., RARS, PSSR, CRI, PRI) combine a carotenoid-sensitive and an insensitive band through ratios or normalized differences, while three-band models use two carotenoid-sensitive and one insensitive band. Reflectance backscattering is addressed in indices such as SIPI and mCRI, which balance pigment-sensitive and insensitive bands.

In addition to the developed models, a widely used Vegetation Index (VI) was calculated for comparison. Ratio Analysis of Reflectance Spectra (RARS) evaluates reflectance values at different wavelengths by calculating their ratios, and has been widely applied in vegetation studies for pigment estimation, plant health, biomass, and stress assessment (Gitelson et al., 2003). The Structure Insensitive Pigment Index (SIPI) emphasizes the carotenoid-to-chlorophyll ratio while minimizing canopy structure effects; its values range from 0 to 2, with green vegetation typically between 0.8 and 1.8, making it suitable for stress detection and yield analysis (Bagherzadeh et al., 2021). Blackburn (Rodriguez-galiano et al., 2014; You et al., 2017; Zhou and Li, 2019) compared multiple ratio-based indices (RARSa, b, PSSRa, b, PSNDa, b) and found PSSR and PSND to be the most effective for predicting chlorophyll a, chlorophyll b, and carotenoids. He also reported strong but non-linear (power or exponential) relationships, varying by wavelength, index type, and whether area- or mass-based measurements were used.

Carotenoids, which protect plants from excess light, generally increase under stress, making them reliable stress indicators. The Carotenoid Reflectance Indices (CRI1 and CRI2) quantify the carotenoid-to-chlorophyll ratio, with values typically between 1 and 12 for green vegetation and up to >15 in stressed plants (Gitelson et al., 2002). CRI2, an adjusted form of CRI1, provides better sensitivity under high carotenoid concentrations. The Modified Carotenoid Reflectance Index (mCRI) further improves sensitivity, particularly in high-carotenoid vegetation, and effectively differentiates healthy from stressed plants affected by drought or disease (Gitelson et al., 2006). The Photochemical Reflectance Index (PRI), sensitive to xanthophyll cycle pigments, reflects photosynthetic light-use efficiency and vegetation stress responses, with applications in monitoring productivity and ecosystem health across crops, forests, and shrublands (**Table 1**) (Guo et al., 2021).

**Table 1:** Vegetation indices used in practice to determine carotenoid estimates.

Model	Type	Bands Used	Key Features	Index	Applications
Two-band models (RARS, PSSR, CRI, PRI)	Ratio/NDVI-type	1 carotenoid-sensitive + 1 insensitive band	Capture pigment absorption patterns; simple ratio-based structure		Carotenoid and chlorophyll estimation (Gamon et al., 1990; Smith, 2020)
Three-band models (e.g., SIPI, mCRI)	Ratio/adjusted ratio	2 carotenoid-sensitive + 1 insensitive band	Correct canopy structure and backscattering effects		Precision monitoring of pigment dynamics (Smith, 2020)
RARS (Ratio Analysis of Reflectance Spectra)	Ratio	Selected reflectance bands	Highlights spectral absorption features of pigments	R_760_/R_500_	Plant health, biomass, stress monitoring (Gitelson et al., 2003)
SIPI (Structure Insensitive Pigment Index)	Ratio	Carotenoid: chlorophyll bands	Index range 0–2; green vegetation typically 0.8–1.8	(R_800_-R_445_)/(R_800_-R_680_)	Stress and canopy structure analysis (Bagherzadeh et al., 2021)
PSSR/PSND	Ratio	Narrow pigment-sensitive bands	High predictive power for chlorophyll and carotenoids	R_800_/R_500_	Remote sensing of pigment content (Rodriguez-galiano et al., 2014; You et al., 2017; Zhou and Li, 2019)
CRI1/CRI2 (Carotenoid Reflectance Indices)	Ratio	Visible spectrum reflectance	Index range 0–15; typical vegetation 1–12	CRI_550_: R_510_^-1^-R_550_^-1^ CRI_700_: R_510_^-1^-R_700_^-1^	Carotenoid-to-chlorophyll ratio; stress detection (Gitelson et al., 2002)
mCRI (Modified Carotenoid Reflectance Index)	Adjusted ratio	Visible spectrum bands	Improved sensitivity in high-carotenoid vegetation	mCRI_G_: (R_510-520_^-1^-R_560-570_^-1^)*R_NIR_ mCRI_RE_: (R_510-520_^-1^-R_690-710_^-1^)*R_NIR_	Differentiates healthy vs. stressed plants (Gitelson et al., 2006)
PRI (Photochemical Reflectance Index)	Reflectance ratio	Xanthophyll-sensitive bands	Indicator of photosynthetic light-use efficiency and stress response	(R_531_-R_570_)/(R_531_+R_570_)	Productivity, stress, ecosystem monitoring (Guo et al., 2021)

A simple linear regression approach was used to develop the carotenoid estimation model by correlating the carotenoid data with the derived indices. The optimal band combinations were determined based on the highest coefficient of determination (R²). For the ML model, the training and testing datasets were randomly sorted before training and testing the developed models, after the cleaning process, to guarantee the evaluation of the performance and accuracy of the models without personal intervention. The accuracy of the predictive models was measured by Root Mean Square Error (RMSE), Normalized Root Mean Square Error (NRMSE), Mean Absolute Error (MAE), Mean Bias Error (MBE), Mean Squared Prediction Error (MSPE) and Nash-Sutcliffe Efficiency (NSE).


$$\;R^{2}=1-\;\frac{\sum_{i=1}^{N}{\left(y_{i}-\overset{´}{y_{i}}\right)}^{2}}{\sum_{i=1}^{N}{\left(y_{i}-\bar{y}\right)}^{2}}$$

$$RMSE=\sqrt{\frac{\sum_{i=1}^{n}{\left(y_{i}-\overset{´}{y_{i}}\right)}^{2}}{n}}$$

$$\text{NRMSE}=\frac{\sqrt{\frac{\sum_{i=1}^{n}{\left(y_{i}-\overset{´}{y_{i}}\right)}^{2}}{n}}}{\left(\bar{y}\right)}100$$

$$MAE=\;\frac{1}{n}\sum_{i=1}^{N}\left|\overset{´}{y_{i}}-yi\right|$$

$$MBE=\;\frac{1}{n}\sum_{i=1}^{N}\left(yi-\overset{´}{y_{I}}\right)$$

$$NSE=1-\frac{\sum_{i=1}^{n}{\left(\overset{´}{y_{i}}-yi\;\right)}^{2}}{\sum_{i=1}^{n}{\left(\overset{´}{y_{i}}-\bar{y}\;\right)}^{2}}$$

where:

*y*_*i*_ : estimated value;

ý_1_ : measured value;

$\bar{\text{y}}$ : mean value of reference samples

n : number of samples used for validation.

### 2.5 Machine learning models for estimating carotenoid content in plants

Although partial least squares regression (PLSR) is frequently applied in spectroscopic studies as a reference method, the primary aim of this study was to explore the potential of machine learning algorithms to capture complex relationships, the analysis was restricted to models that are inherently capable of handling non-linearity and high-dimensional feature spaces. Estimating carotenoid content in plants using machine learning (ML) is an active research area, particularly for precision agriculture, food quality assessment, and plant breeding. Various ML models have been employed for this task, often using spectral data, image analysis, or other sensor-based approaches. The developed machine learning models have already been cross-validated five times. In this approach, the entire dataset is divided into five parts; in each iteration, four parts are used for training and one for verification, which is repeated five times to test all the data. This ensures robust and generalized performance across different years, eliminating the need for separate annual breakdowns.

#### 2.5.1 Random forest

Random Forest is a supervised learning algorithm that constructs an ensemble of decision trees, often using the bagging method. Bagging, or bootstrap aggregating, is based on the principle that combining multiple learning models enhances overall performance and accuracy. Random Forest (RF) is an ensemble learning technique that generates multiple decision trees (DT) by selecting subsets of samples from the original dataset using the bootstrap method (Song et al., 2020). Once the ensemble classifier is built and finalized, the predictions from individual trees are aggregated by majority voting for classification tasks or by averaging for regression tasks to produce the final RF prediction (Cipullo et al., 2019). Additionally, pruning techniques can be applied to enhance predictive performance by reducing tree complexity, particularly when large, intricate trees arise due to dataset size (Pérez et al., 2021). RF demonstrates exceptional performance when handling large, high-dimensional, noisy, and imbalanced datasets while effectively mitigating overfitting (Cruz et al., 2022). Decision trees within RF can quickly learn and process both categorical and numerical data with minimal preprocessing, as they do not require assumptions about data properties such as linearity or normality (Breiman, 2001; Barmpalias and Wang, 2021). Furthermore, RF provides valuable insights into the importance of input variables, offers greater tolerance for missing data, and outperforms many other methods in terms of robustness and interpretability (Song et al., 2008; Pham et al., 2019). For building this model, some parameters were identified: A user-defined number of trees (default = 100); Each tree is trained on a bootstrap sample of the data; At each node in a tree, it selects the best split among a random subset of attributes (n/3 for regression by default). The Random Forest model was chosen to handle the high dimensionality of hyperspectral data and accurately reveal nonlinear relationships. The equation used in the model is shown as follows:


$$RF\_\mathrm{Pr}edict\left(x\right)=\left(1/k\right)*\sum h_{i}\left(x\right)$$

Where: Each h_i_(x) returns a numeric prediction. The final prediction is the average of all tree predictions.

#### 2.5.2 REPtree – reduced error pruning tree

The REP Tree is a decision tree learner that builds a tree using information gain and prunes it using reduced-error pruning. The REPtree combines the Reduced Error Pruning (REP) method with the fast decision tree learning algorithm, which incorporates both splitting and pruning steps (Quinlan, 1987). This approach applies the decision tree algorithm to simplify the modeling process using a training dataset, particularly when the output of the tree is large, and REP is employed to reduce the complexity of the tree’s structure (Mohamed et al., 2012). The pruning step in the REPT algorithm addresses the issue of backward overfitting. The REPT algorithm aims to identify the smallest, most accurate sub-tree through a post-pruning technique. The model’s performance is determined by the information gain from entropy or the reduction in variance, coupled with the reduced error pruning techniques (Srinivasan and Mekala, 2014). REPtree was used for its simplicity and reduction of overfitting, while maintaining predictive accuracy. REP Tree does not produce a mathematical equation, but instead generates a tree of if-then rules, as follows:

att1<= VALUE.

att2 = high: Class A.

att2 = low: Class B.

att1 > VALUE: Class C.

Where x1=att1, x2=att2.

Conditions are based on attributes (att1, att2); Leaves show class labels and instance counts (e.g., class1 (30.0) means 30 instances in this class at that leaf). For regression: leaves contain predicted values.

#### 2.5.3 Random subspace

The Random Subspace method is an ensemble learning technique, like Bagging or Random Forest but it works by training each base learner on a random subset of attributes (features), rather than a random subset of data samples. The RS is an ensemble and parallel learning algorithm. In this method, multiple decisions from classifiers are combined by optimizing subsets. These subsets are randomly chosen from the feature space of training classifiers. What distinguishes the RS from other ensemble algorithms is that it is applied to different samples (Skurichina and Duin, 2002). In the first stage, the original feature space is classified into L training subsets, each with q-dimensionality. A base classifier is then applied to each subset using the REPT algorithm. Ultimately, the final decision, which combines the results from the base classifiers, is made based on weighted majority voting (Sugasawa, 2017). The Random Subspace method was chosen to analyze subsets of spectral variables and increase the robustness of the model. The conceptual equation of RS model is:


$$Prediction\left(x\right)=\;1/k\sum_{i=1}^{k}C_{i}\left(x\right)$$

Where x = new input instance; Each Ci is trained on: The full training data (rows) and random subset of features (columns). k=number of individual models in the ensemble.

#### 2.5.4 Bagging

Bagging (Bootstrap Aggregating) is a meta-classifier that builds an ensemble of base learners (typically decision trees like J48) trained on random subsets (with replacement) of the training data. Bootstrap aggregating, commonly referred to as bagging, is a powerful ensemble learning technique that enhances the accuracy and robustness of predictive models in machine learning. The fundamental principle of bagging involves two key processes: bootstrapping and aggregating. Bootstrapping entails generating multiple datasets through random sampling with replacement from the original dataset, allowing for the creation of diverse training sets for model training. This diversity is crucial as it enables the individual models to learn different aspects of the data, thereby reducing variance and improving overall model performance (Helmut and Murdiansyah, 2023). The effectiveness of bagging is particularly evident in its ability to mitigate overfitting, which is a common issue in complex models. By averaging the predictions of several models, bagging smooths out the noise and reduces the likelihood of fitting to random fluctuations in the training data (Gitelson and Solovchenko, 2017). Research has shown that bagging can significantly improve the performance of various machine learning algorithms, including decision trees and support vector machines. For instance, studies have demonstrated that bagging applied to decision trees can lead to substantial gains in accuracy, especially in scenarios with high variance (Huang et al., 2018). Bagging does not produce a single algebraic equation but instead combines predictions from multiple models. Process is built by creating number of base learners (k) bootstrap samples (random samples with replacement) from the training data then Training k base classifiers, one on each bootstrap sample and Predict using average prediction. Machine learning models have been implemented in Waikato Environment for Knowledge Analysis (WEKA), which is Java-based and includes a GUI, command-line interface, and Java API. It is a popular open-source software for machine learning and data mining. Bagging was used to improve predictive accuracy and increase model stability based on spectral indices.

Tree 1: IF age<= value THEN class = Yes ELSE class = No.

Tree 2: IF income > value THEN class = Yes ELSE class = No.

Final Prediction: Majority vote across k trees.

In our study, model parameters were optimized using the standard procedures available within the WEKA platform. Specifically: Random Forest was tuned by setting the number of trees (100) and applying random feature subsets at each split; REPTree applied reduced-error pruning to limit tree complexity; Bagging and Random Subspace relied on bootstrap sampling and random feature subsets. These methods inherently include mechanisms to reduce overfitting by pruning or aggregating multiple learners. In addition, the dataset was divided into 70% training and 30% testing, and we performed cross-validation to ensure the stability of the models and to prevent bias from a single train–test split.

## 3 Results

### 3.1 Maize carotenoid content and leaf reflectance % results

**Figure 2:**
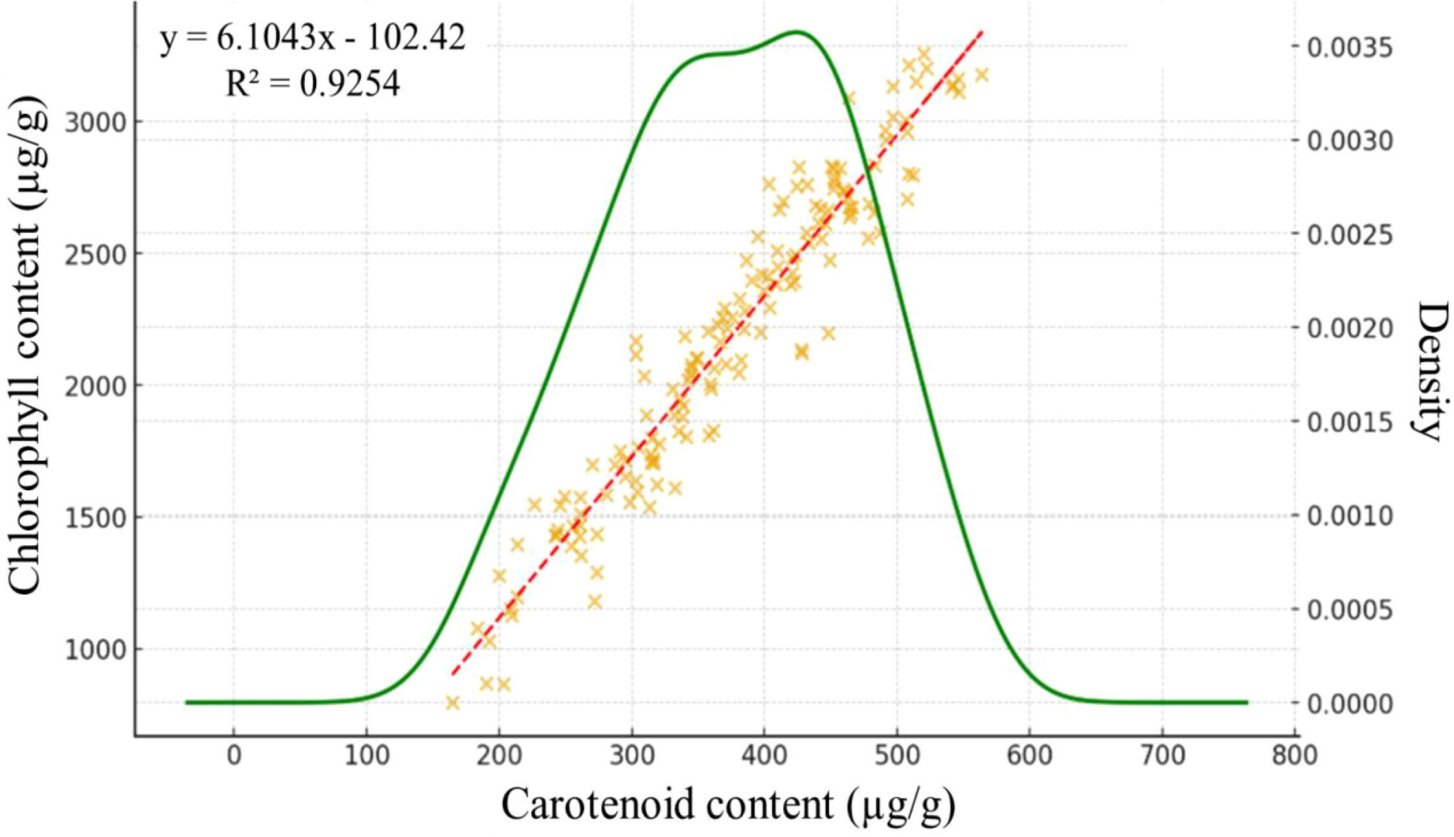
Correlation between carotenoid and chlorophyll content.

**Figure 2** illustrates the relationship between carotenoid and chlorophyll content in plant samples. From the data it is clear that there is a strong positive linear relationship between the two parameters, as confirmed by the regression line fitted to the scatter plot. The strength of the relationship is also supported by the coefficient of determination (R² = 0.925), which shows that carotenoid content can explain the variation in chlorophyll content in more than 92% of cases. This high value indicates an excellent model fit. Chlorophylls are primarily responsible for light harvesting and photosynthetic capacity, whereas carotenoids serve both as accessory pigments in light absorption and as protective pigments against photooxidative stress. Thus, carotenoid dynamics can diverge from chlorophyll under specific physiological or environmental conditions, such as high light, drought, or senescence, when photoprotective demands change independently of chlorophyll levels. Measuring chlorophyll content alone may therefore capture photosynthetic capacity, but carotenoid estimation provides complementary information on photoprotection, stress response, and pigment balance. In this context, the high correlation reflects shared regulation under the studied conditions, but under stress scenarios the two traits may decouple, making carotenoid monitoring particularly valuable.

**Figure 3:**
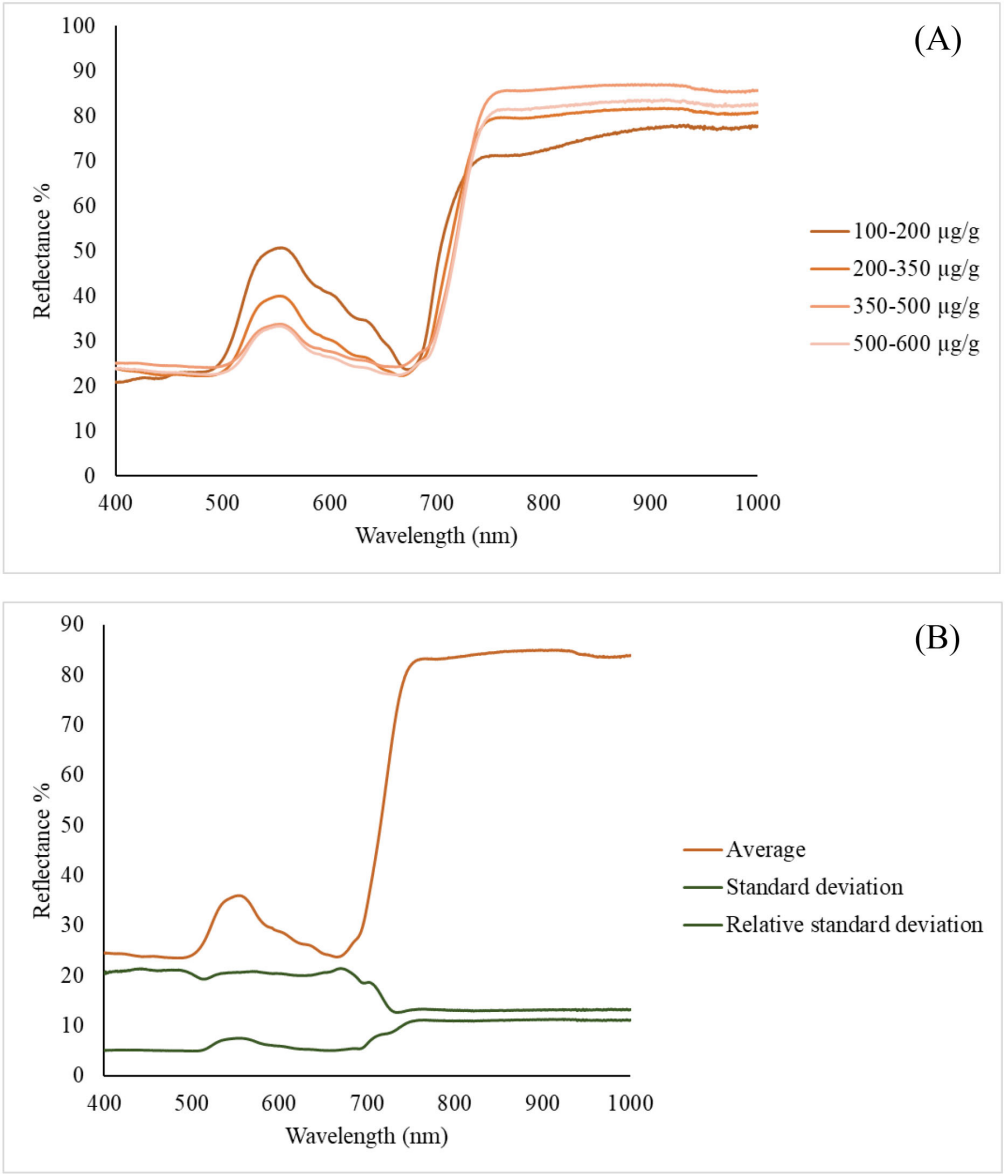
Maize leaf carotenoid content reflectance % values **(A)**, Maize leaf carotenoid content reflectance % values statistical **(B)**.

The spectral reflectance of maize leaves was evaluated in the wavelength range 400–1000 nm at four different carotenoid concentration ranges (100-200 µg/g, 200-350 µg/g, 350-500 µg/g, and 500-600 µg/g). Marked differences were observed in the visible spectrum (especially in the region 500–650 nm) between samples with different carotenoid contents. Carotenoids, such as lutein and β-carotene, characteristically absorb blue (450–500 nm) and green (500–570 nm) wavelengths of light, and their presence reduces leaf reflectance in these regions. Accordingly, lower reflectance values are observed for samples with higher carotenoid concentrations, while lower concentrations have higher reflected light intensities. A sharp increase in reflectance is observed in the 680–700 nm range of the spectrum, corresponding to the so-called “red edge” phenomenon. This reflects the state of the plant’s photosynthetic apparatus and is an important indicator of the physiological activity of the leaf. Above 700 nm, the reflectance is highly stabilised in the NIR range, which is mainly determined by the structural features of leaf tissues, such as cell walls and intercellular air spaces. In this region, the carotenoid content has less influence on spectral reflectance, but slight differences can be observed, which may indicate differences in leaf structure between samples from different regions. Overall, the figure shows that the carotenoid content of maize leaves has a significant effect on the spectral reflectance characteristics, especially in the visible range. The use of this type of spectral analysis offers the possibility of non-destructive estimation of leaf pigment composition, in particular the amount of carotenoids, based on remote sensing. The lowest carotenoid content was 114.72 µg/g and the highest carotenoid content was 526.97 µg/g. The average carotenoid content is 382.31 µg/g ± 86.02 µg/g (**Figure 3**).

### 3.2 Testing of available carotenoid indeces

**Figure 4:**
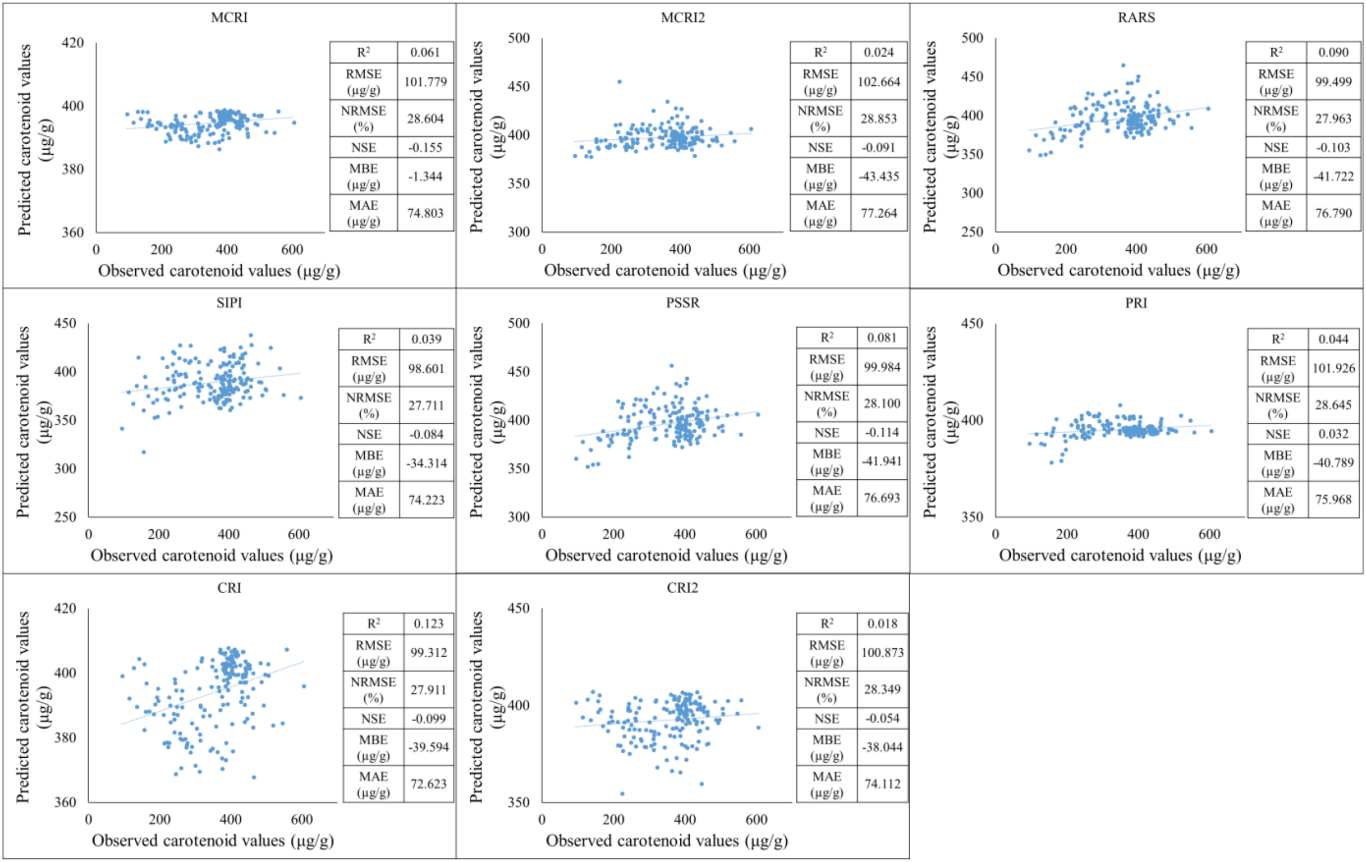
Correlations between observed and predicted values with vegetation indices used in practice.

Based on the measured spectral data, models for estimating carotenoid content already used in practice were tested, but the results showed that the predictive accuracy of these models was not adequate for the samples we investigated. The R^2^ values range from 0.039 to 0.123, indicating that none of the models explain the variance of the data well. The CRI model performs the best with 0.123, while the SIPI model performs the worst with 0.039. RMSE values range from 98.601 µg/g to 101.926 µg/g. Lower values indicate better accuracy. The SIPI model shows the best performance with 98.601 µg/g while the PRI model shows the worst with 101.926 µg/g. NRMSE values show small differences between 27.711% and 28.645%, indicating that the variation between models in this respect is minimal. NSE all models have a negative NSE value (except PRI: 0.032), suggesting that the models do not perform better than a simple average-based forecast. MBE values range widely from -1.344 µg/g to -41.941 µg/g. The MCRI model has the smallest absolute deviation of -1.344 µg/g, while the PSSR shows the largest negative value -41.941 µg/g, indicating significant underestimation. MAE values range from 72.623 µg/g to 76.790 µg/g. Based on the analysis, none of the models perform exceptionally well, as R² values are low and NSE indicators are mostly negative. The CRI model shows the best overall performance, as it has the highest R² value and one of the lowest MAE values. The worst performance is shown by the SIPI, which has the lowest R² value but a more favourable RMSE (**Figure 4**).

### 3.3 Identification and construction of new carotenoid indices based on PCA

Principal component analysis produces five principal component results that capture the variance of the data. The first component explains 78.203% of the total variance, which means that it captures most of the information in the dataset. Component 2 adds a further 14.408%, bringing the cumulative explained variance to 92.611%. Component 3 adds 4.157%, resulting in a cumulative total explained variance of 96.768%. Component 4 increases the explanation by 2.639%, reaching 99.407%. Component 5 explains only 0.289%, resulting in a cumulative variance of 99.696%. Since Component 1 captures most of the variance (78.203%) and Component 2 increases this to 92.611%, these two components alone are sufficient to represent the bulk of the data structure (**Table 2**). This would significantly reduce the complexity from five dimensions to only two without a large loss of information. To test each variable of the measured sample, we used the Kaiser–Meyer–Olkin (KMO) test. The KMO was 0.84, indicating that the PCA is in the middle and demonstrating that the sampling is adequate (**Table 2**).

**Table 2:** Results of the principal component analysis.

Component	% of Variance	Cumulative%
1	78.203	78.203
2	14.408	92.611
3	4.157	96.768
4	2.639	99.407
5	0.289	99.696

**Figure 5:**
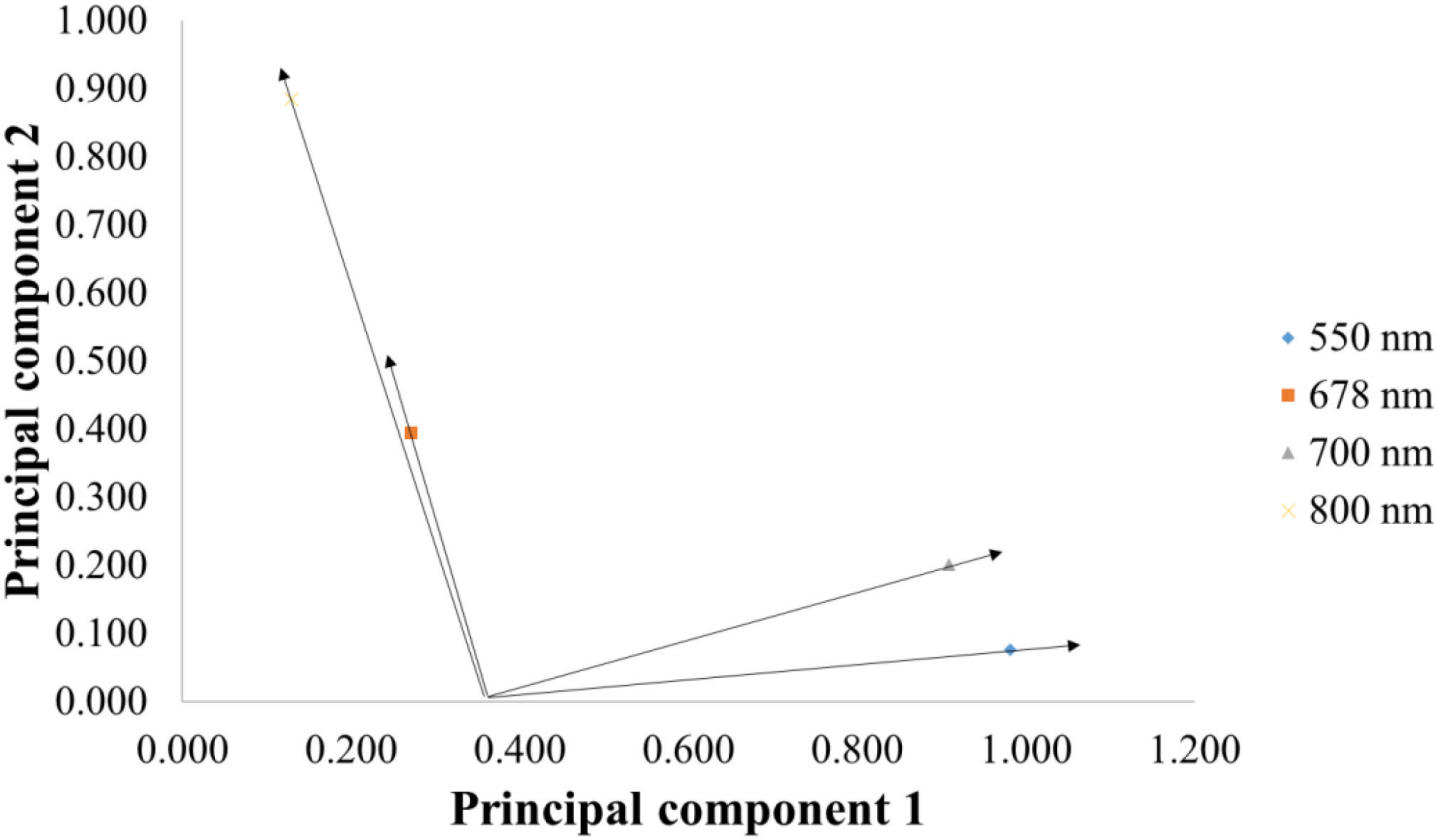
Two-dimensional PCA matrix (PC1 × PC2) for the used wavelength bands.

To analyse the role of bands in pigment sensitivity, a PC1-PC2 plot was also produced. Since the total number of variables is 1063, interpretation of the PCA matrix (PC1 × PC2) plot of the variables would be very crowded, so only the 4 bands were placed on the plot. Based on the PC1-PC2 plot, the 800 nm wavelength vector is moderately correlated with PC2, as it is mainly located along the y-axis. Meanwhile, in the case of carotenoid, the 550 nm and 700 nm wavelengths contribute less to PC2 and are more associated with PC1. The 678 nm wavelength, however, is less strongly associated with the first principal component, as its vector is short and tends to point along the first component. The 700 nm direction vector is longer and along the first component, indicating a strong coupling. The carotenoid vector is almost entirely in the negative region of the x-axis, indicating that the carotenoid content is more strongly coupled to PC1, but shows weaker correlation (**Figure 5**).

**Figure 6:**
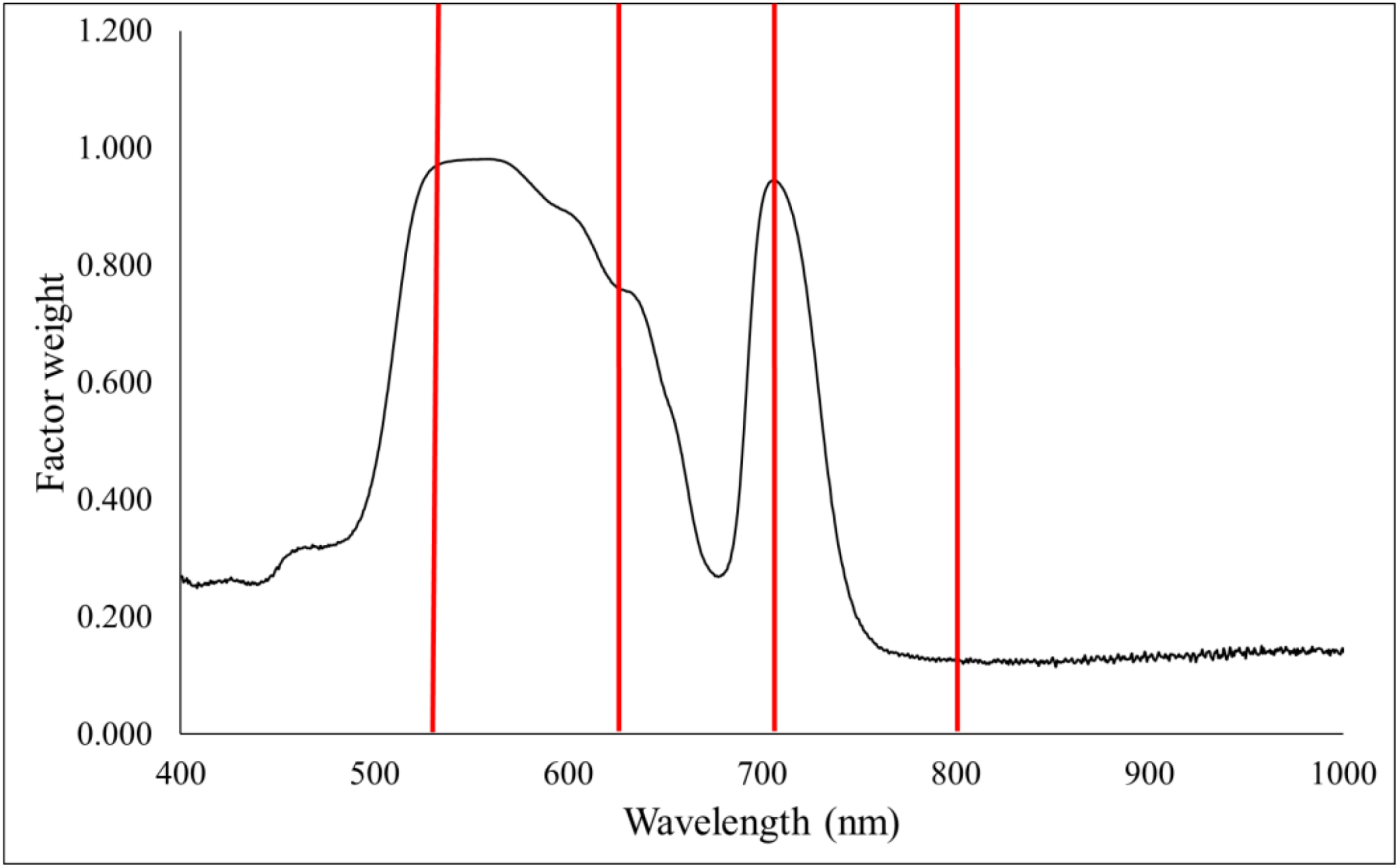
Principal component analysis of carotenoid values.

Principal component analysis resulted in 5 principal components. Between 500–700 nm, a significant increase is observed, peaking at two peaks. After 700–800 nm, the curve returns to a lower, stable value, and only minimal fluctuations are observed; thus, the wavelength ranges 550 nm, 678 nm, 700 nm, and 800 nm were selected to build the carotenoid estimator models (**Figure 6**).

In the factor weight were observed, of which the 800 nm range was used together with the 550 nm, 678 nm and 700 nm ranges to construct the carotenoid estimation indices. Based on the principal component analysis, nine new indexes for estimating maize chlorophyll content were created. Nine indices were developed using the results of principal component analysis and standard deviation:


$$\text{CA}{\text{R}}_{1}={\text{R}}_{700}/{\text{R}}_{678}$$

$$\text{CA}{\text{R}}_{2}={\text{R}}_{700}/{\text{R}}_{800}$$

$$\text{CA}{\text{R}}_{3}=\left({\text{R}}_{550}+{\text{R}}_{700}\right)/{\text{R}}_{678}$$

$$\text{CA}{\text{R}}_{4}=\left({\text{R}}_{550}+{\text{R}}_{700}\right)/{\text{R}}_{800}$$

$$\text{CA}{\text{R}}_{5}={\text{R}}_{550}/{\text{R}}_{678}$$

$$\text{CA}{\text{R}}_{6}={\text{R}}_{550}/{\text{R}}_{800}$$

$$\text{CA}{\text{R}}_{7}=\left({\text{R}}_{550}+{\text{R}}_{700}\right)/\left({\text{R}}_{678}+{\text{R}}_{800}\right)$$

$$\text{CA}{\text{R}}_{8}={\text{R}}_{700}/\left({\text{R}}_{678}+{\text{R}}_{800}\right)$$

$$\text{CA}{\text{R}}_{9}={\text{R}}_{550}/\left({\text{R}}_{678}+{\text{R}}_{800}\right)$$

### 3.4 New carotenoid models calibration and validation

**Figure 7:**
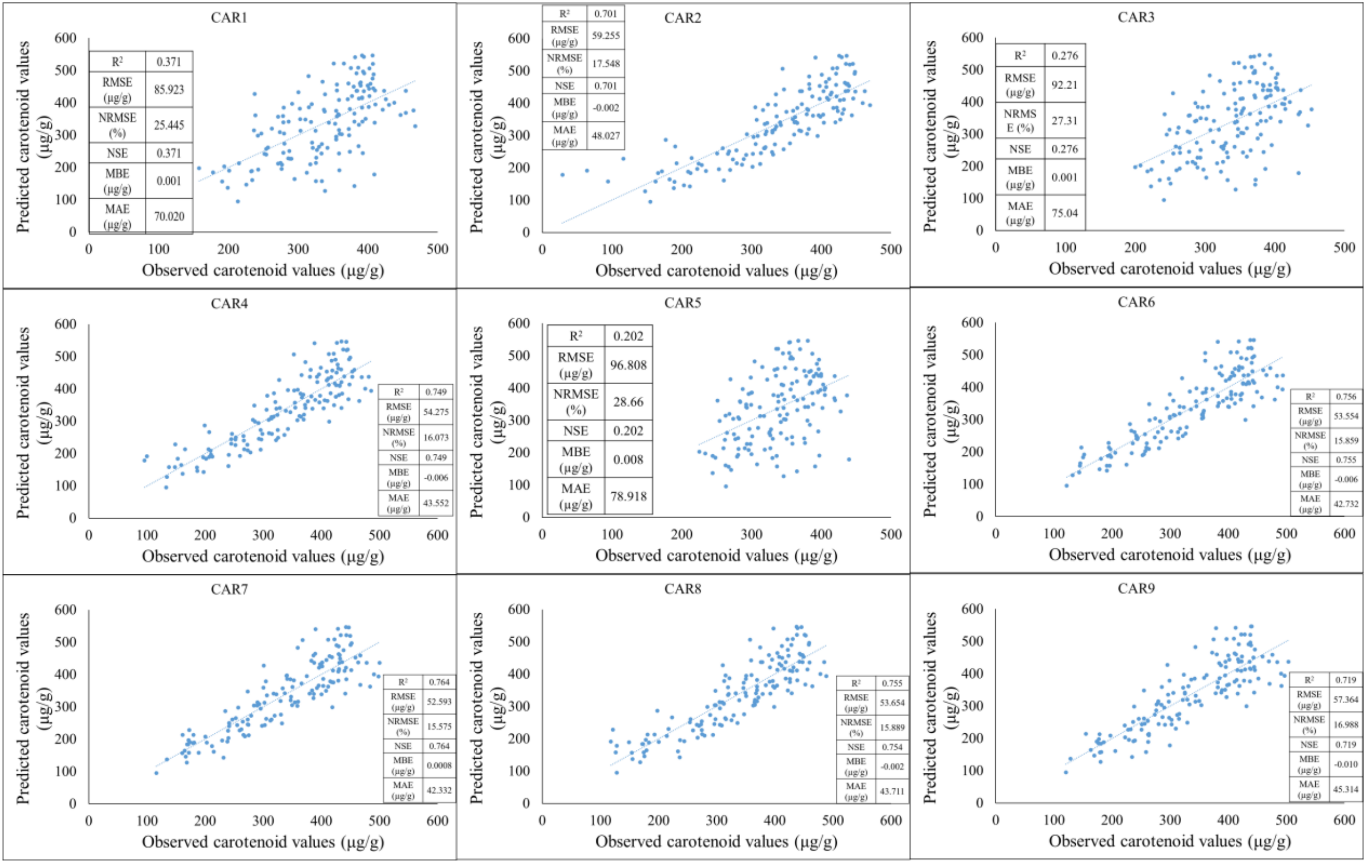
The results of the new model lead to the development of carotenoid predictions during the training period (2021-2022).

The models were compared based on the years of the study and their applicability during training and testing periods was observed. First, the training period was based on data from 2021-2022, while the testing period was based on data from 2023. Based on the results of the training period, the predictive performance of the models shows significant differences for the individual carotenoid fractions. The strongest linear relationship between observed and estimated values was observed for the CAR_7_ variable, which was characterized by a high R² value, low NRMSE, and high NSE, indicating the model’s excellent explanatory and predictive power. The CAR_9_ and CAR_2_ models showed similarly good performance, with point clouds closely following the regression line and error values remaining moderate. These results suggest that the estimation of these carotenoid components by spectral or predictor variables is stable and reliable. In contrast, the CAR_1_, CAR_3_, and CAR_5_ models showed lower coefficients of determination and higher normalized error values, indicating a weak relationship between the observed and predicted values. For these variables, the point clouds showed significant dispersion, and the regression lines described the variability of the data only to a limited extent, suggesting that the model used or the predictor variables included are not able to adequately capture the variability of these carotenoid fractions. The CAR_4_ and CAR_6_ variables showed moderate performance: the R² values indicated moderate explanatory power, while the NRMSE and RMSE values indicated acceptable but not outstanding accuracy. These models showed slight underestimation in the higher concentration ranges, as indicated by negative MBE values, but the degree of bias can be considered negligible overall (**Figure 7**).

**Figure 8:**
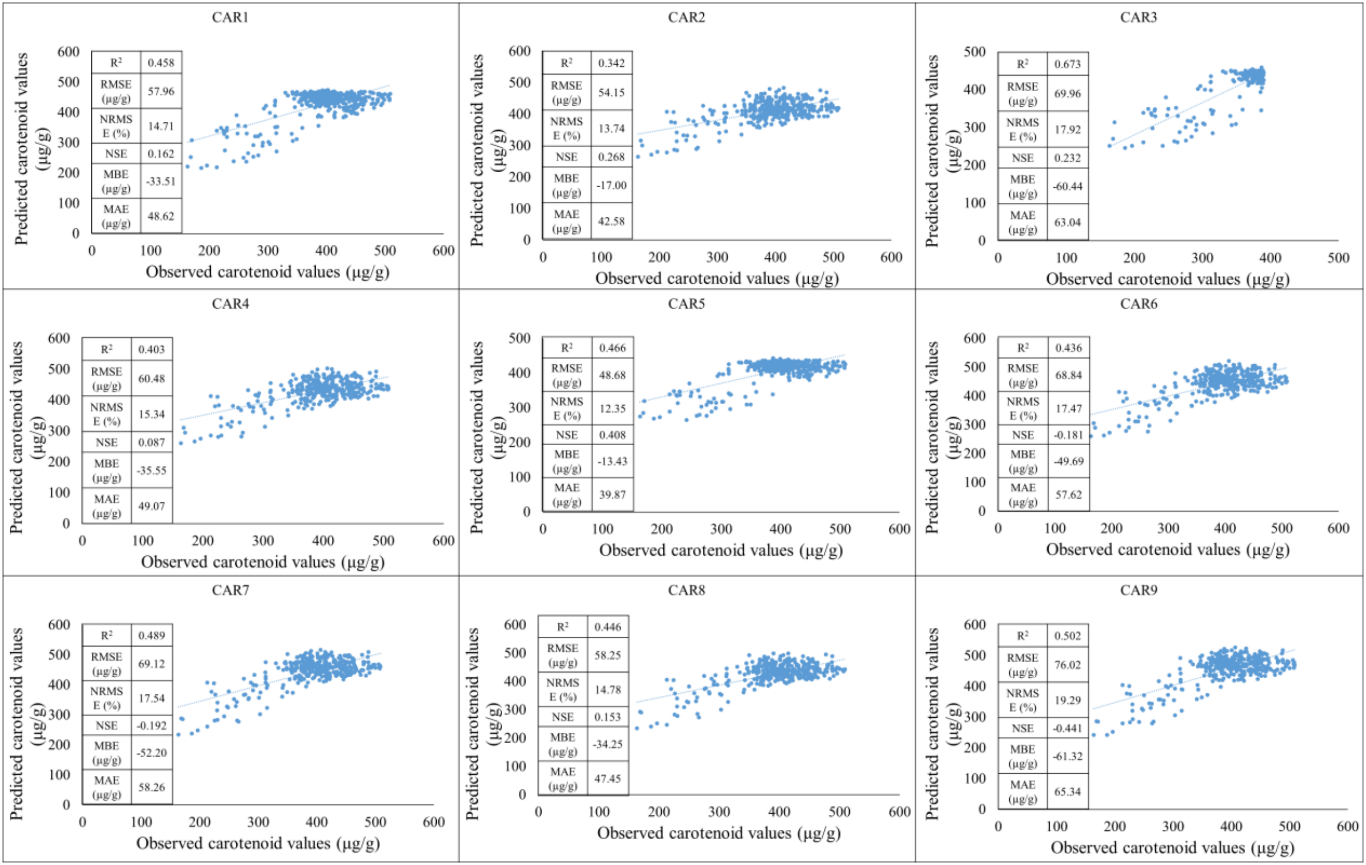
The results of the new model lead to the development of carotenoid predictions during the testing period (2023).

During the testing period, a moderate positive linear relationship can be observed between the observed and estimated values in the CAR_1_ model. The coefficient of determination indicates a moderately high value, suggesting that the model is able to explain a significant portion of the variance. At the same time, based on the RMSE and NRMSE values, the estimation error is not negligible, especially in the lower value range, where the standard deviation of the point cloud increases. The CAR_2_ model shows a poorer fit. In the case of the CAR_3_ model, the statistical indicators show stronger performance. The higher R² and lower normalized error values are consistent with the visually observable prediction. The CAR_4_-CAR_8_ model provides moderately good predictive performance. The performance of the CAR_9_ model is favorable, showing a stronger correlation than the other models set up (**Figure 8**).

**Figure 9:**
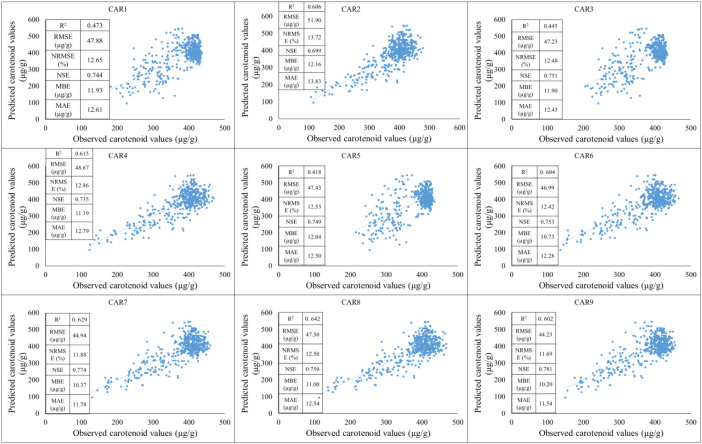
The results of the new model lead to the development of carotenoid predictions during the training period (2021-2023).

Secondly, the training period was based on data from 2021–2023, while the testing period was based on data from 2022. Based on the results of the training period, the predictive performance of the models shows significant differences for each carotenoid fraction. The strongest linear relationship between observed and estimated values was observed for the CAR_8_ variable, which was characterized by a high R² value, low NRMSE, and high NSE, indicating the model’s excellent explanatory and predictive power. The CAR_7_ and CAR_4_ models showed similarly good performance, with point clouds closely following the regression line and error effects remaining moderate. These results suggest that the estimation of these carotenoid components by spectral or predictor variables is stable and reliable. In contrast, the CAR_1_, CAR_3_, and CAR_5_ models showed lower coefficients of determination and higher normalized errors, indicating a weak relationship between observed and estimated values. For these variables, the point clouds showed significant dispersion, and the regression lines described the variability of the data only to a limited extent, suggesting that the model used or the predictor variables are not able to adequately map the variability of these carotenoid fractions (**Figure 9**).

**Figure 10:**
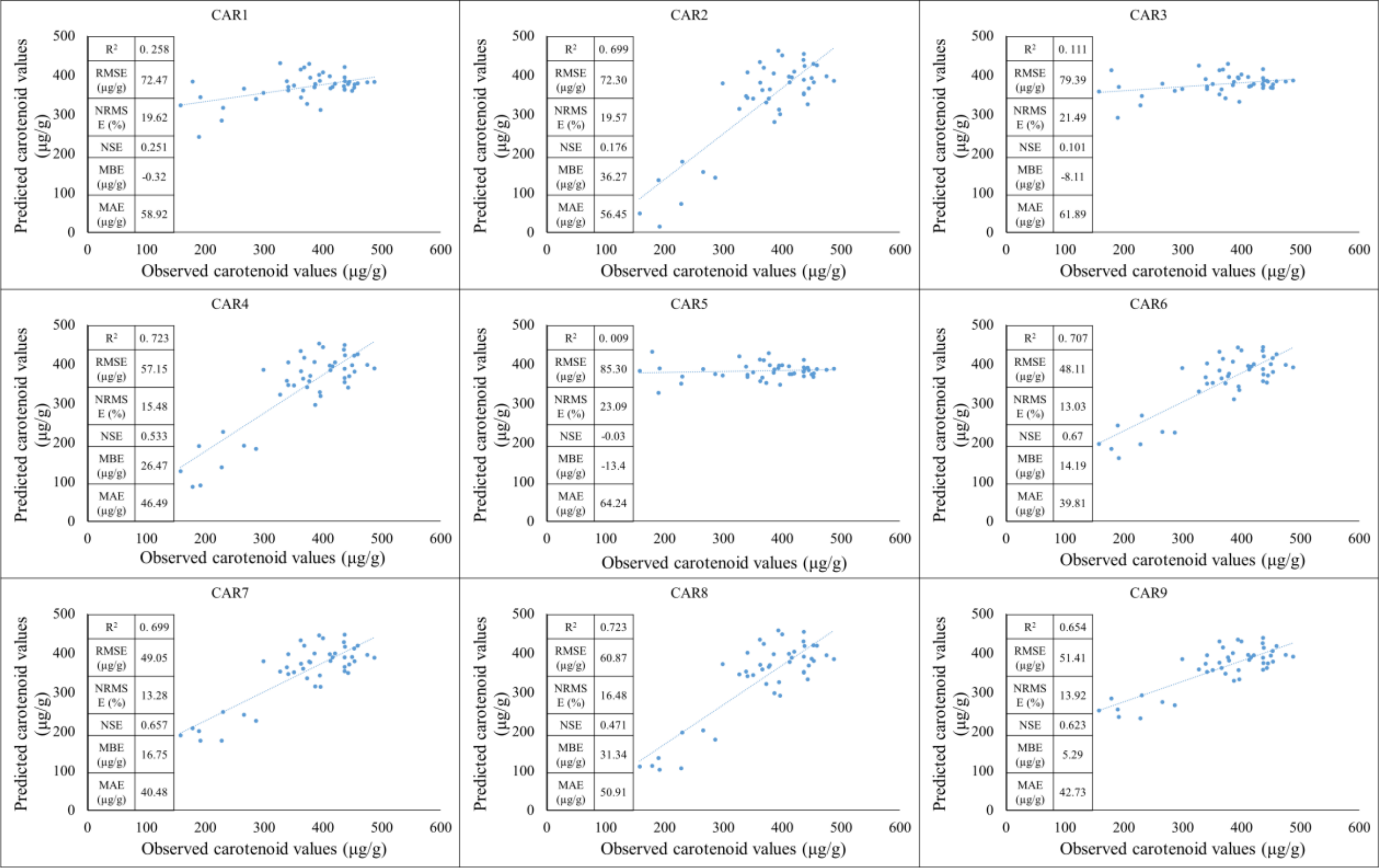
The results of the new model lead to the development of carotenoid predictions during the testing period (2022).

During this testing period, we observed better results than in the previous one. In the case of the CAR_2_, CAR_4_, CAR_6_, CAR_7_, CAR_8_, and CAR_9_ models, a strong positive linear relationship can be observed between the observed and estimated values. The coefficient of determination indicates a high positive value, suggesting that the model can explain a significant portion of the variance. At the same time, based on the RMSE and NRMSE values, the estimation error is not negligible. The lowest estimation value can be observed in the CAR_3_ and CAR_5_ models (**Figure 10**).

**Figure 11:**
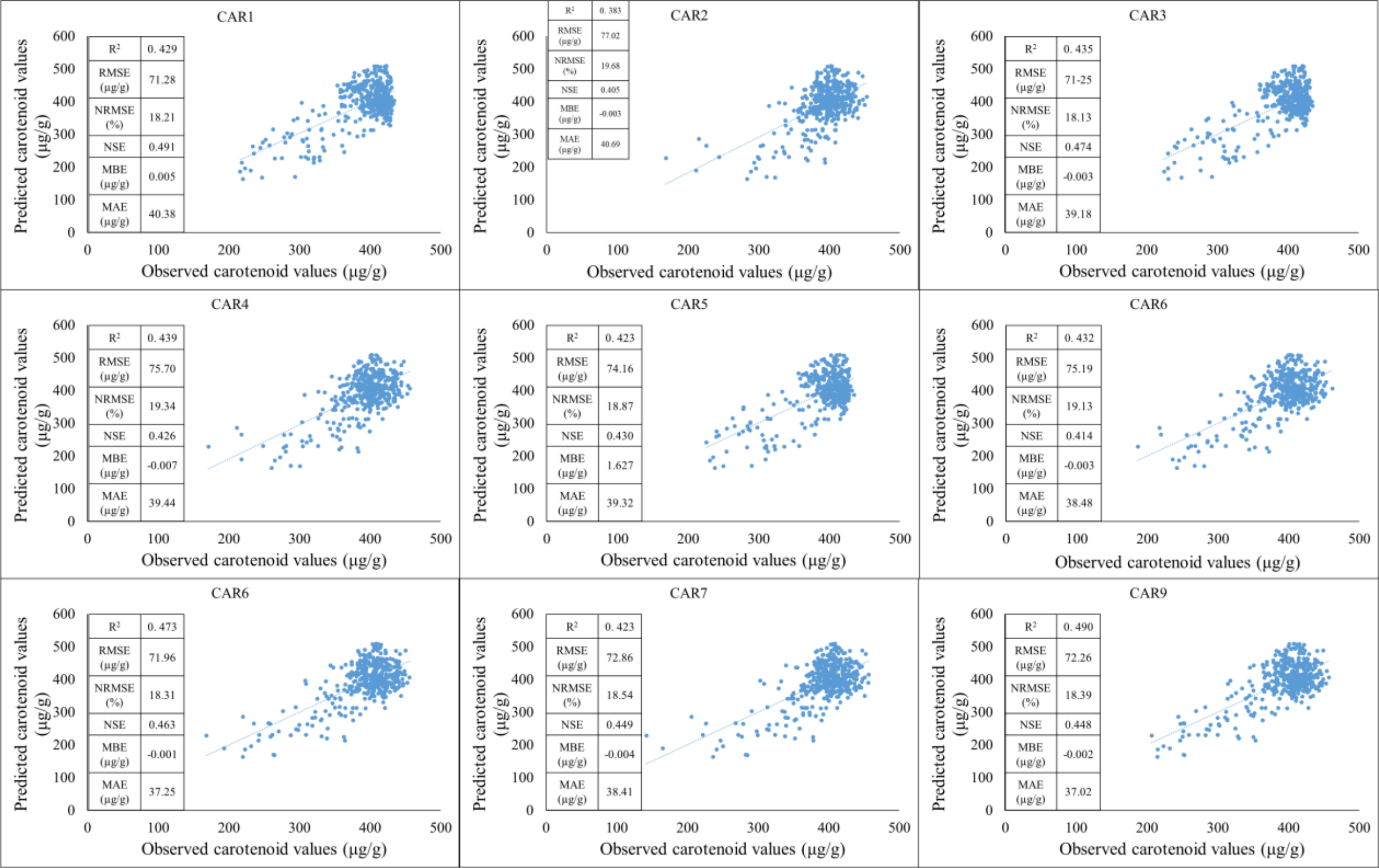
The results of the new model lead to the development of carotenoid predictions during the training period (2022-2023).

For the third time, the training period was based on data from 2022–2023, while the testing period was based on data from 2021. Based on the results of the training period, the predictive performance of the models shows significant differences for each carotenoid fraction. The strongest linear relationship between observed and estimated values was observed for the CAR_9_ variable, which was characterized by a high R² value, low NRMSE, and high NSE, indicating the model’s excellent explanatory and predictive power. In contrast, the other models showed lower coefficients of determination and higher normalized errors, indicating a weak relationship between the observed and estimated values (**Figure 11**).

**Figure 12:**
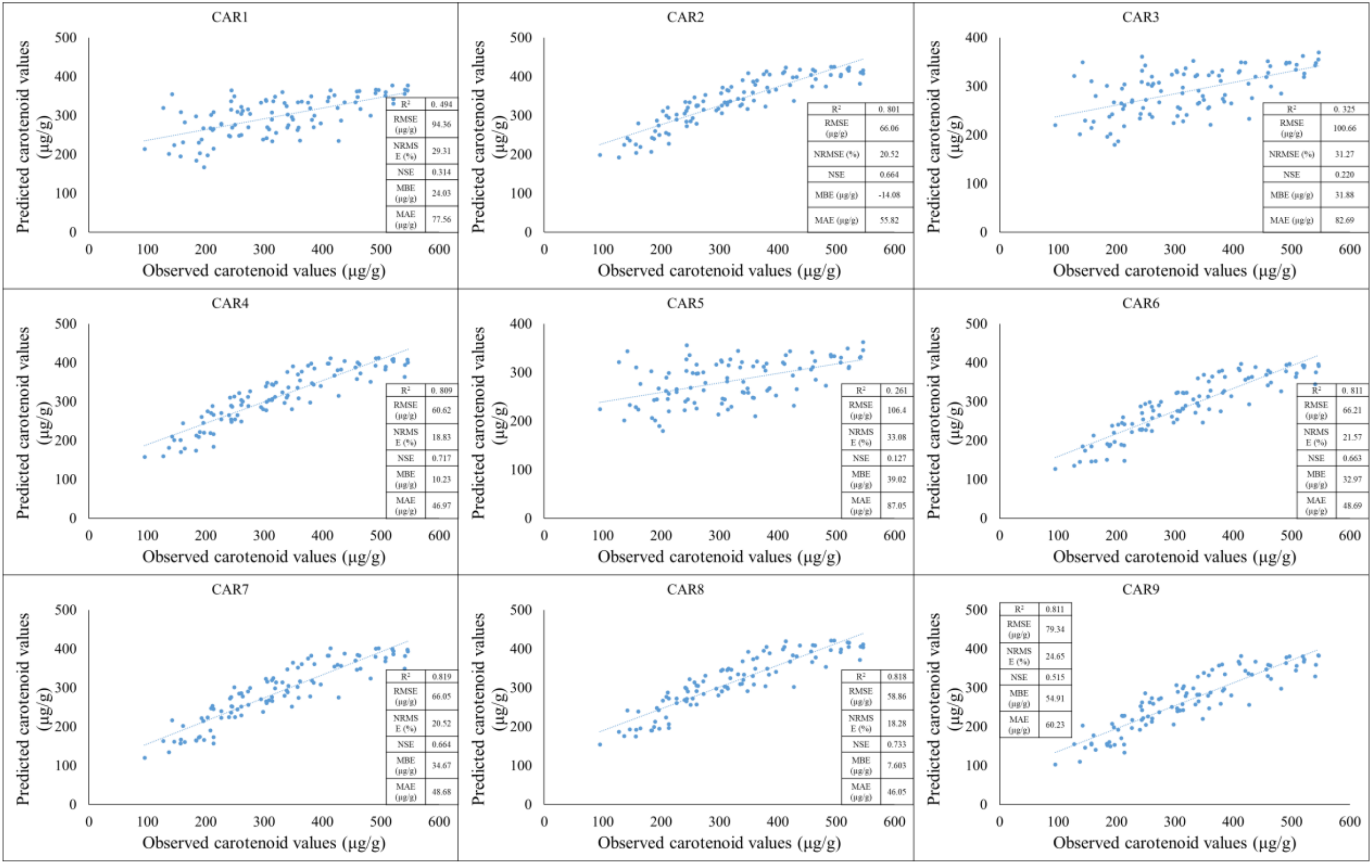
The results of the new model lead to the development of carotenoid predictions during the testing period (2021).

During this test period, we observed better results than in the previous one. In the case of the CAR_2_, CAR_4_, CAR_6_, CAR_7_, CAR_8_, and CAR_9_ models, a strong positive linear relationship can be observed between the observed and estimated values. The coefficient of determination indicates a strong positive high value (above 0.8), which suggests that the model can explain a significant part of the variance. At the same time, based on the RMSE and NRMSE values, the estimation error is not negligible. The lowest estimation value can be observed in the CAR_3_ and CAR_5_ models (**Figure 12**).

**Figure 13:**
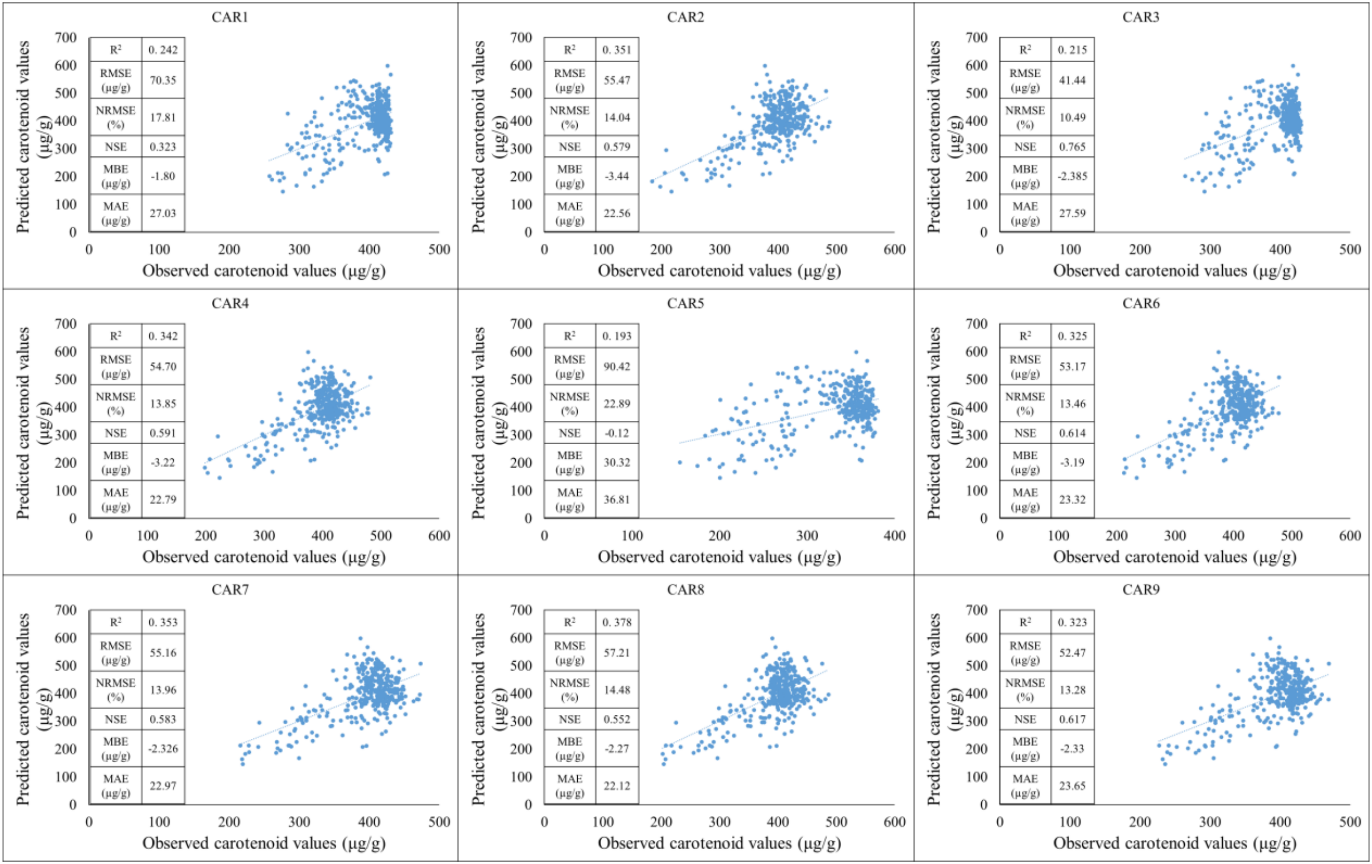
The results of the new model lead to the development of carotenoid predictions during the training period (All datas).

Finally, the data from the years were processed together and then divided into two parts: two-thirds were used to train the models and one-third was used for testing. Based on the results of the training period, the predictive performance of the models shows significant differences for the individual carotenoid fractions. The strongest linear relationship between observed and estimated values was observed for the CAR_2_, CAR_7_, and CAR_8_ variables, which were characterized by moderate R² values, moderate NRMSE, and NSE, indicating the explanatory and predictive power of the model. In contrast, the other models showed lower coefficients of determination and higher normalized errors, indicating a weak relationship between the observed and estimated values (**Figure 13**).

**Figure 14:**
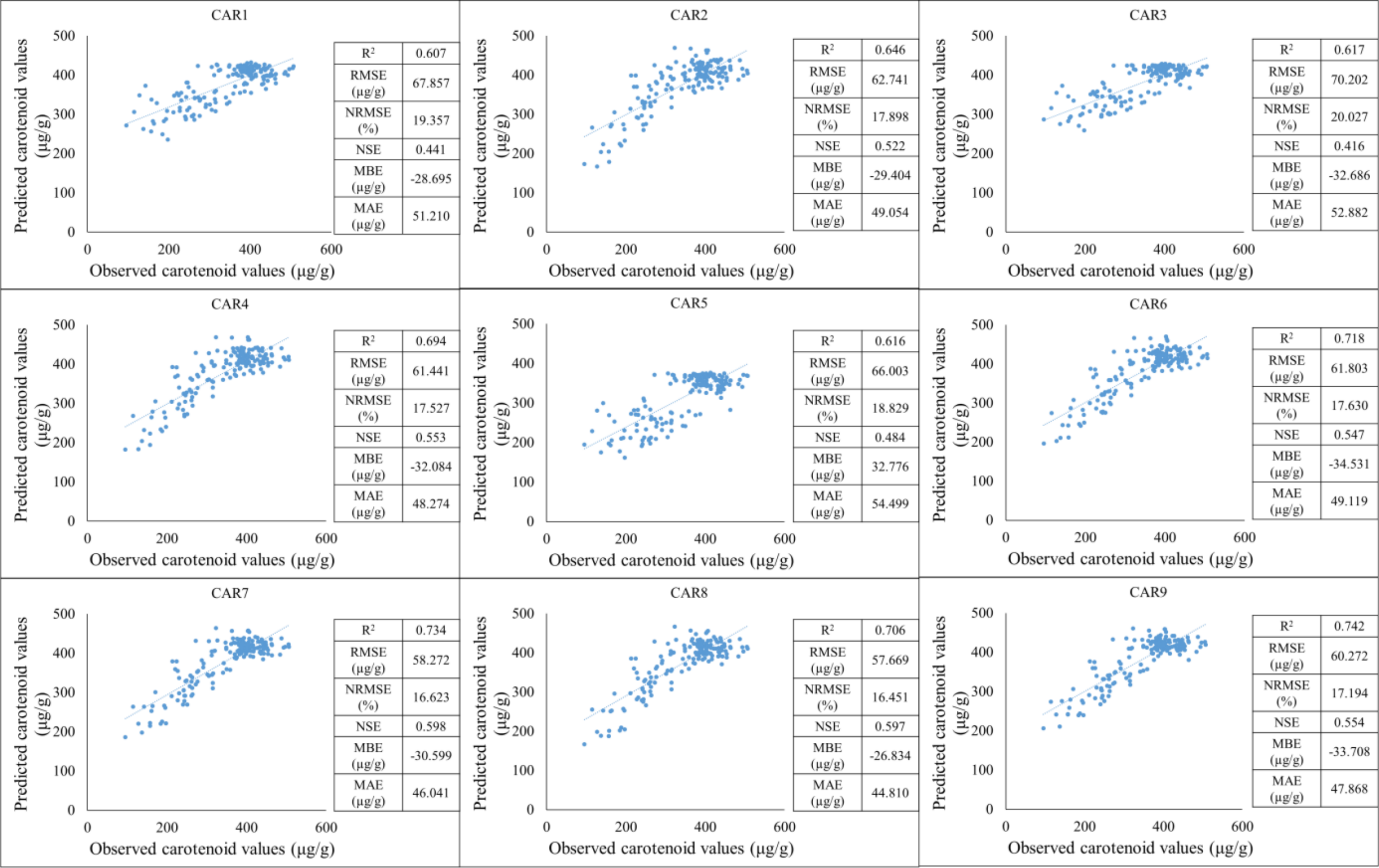
The results of the new model lead to the development of carotenoid predictions during the testing period (All datas).

Based on the testing period, the CAR_9_ model proved to be the best in terms of R² value, suggesting that this model best explains the variability of the target variable. The CAR_1_ model achieved the weakest R² value, indicating lower predictive power. Based on the RMSE, the CAR_8_ model has the smallest average prediction error, while the CAR_3_ model showed the largest error. Similarly, based on the NRMSE and MAE values, the CAR_8_ model performs best, while the CAR_3_ and CAR_5_ models represent weaker performance. The CAR_8_ model showed the smallest systematic bias, while the largest bias was characteristic of the CAR_6_ model. Based on NSE, the CAR_7_ model performs best, while the CAR_3_ model shows the weakest performance. Overall, the CAR_8_ model stands out in terms of RMSE, NRMSE, MBE, and MAE, and is also among the best in terms of R² and NSE. The weaker models are CAR1 and CAR_3_, especially according to the RMSE, NRMSE, and NSE indicators. Based on the above analysis, the CAR_7_ and CAR_8_ models are recommended for further application or development (**Figure 14**).

### 3.5 Carotinoid modeling and testing with ML using new indices

**Figure 15:**
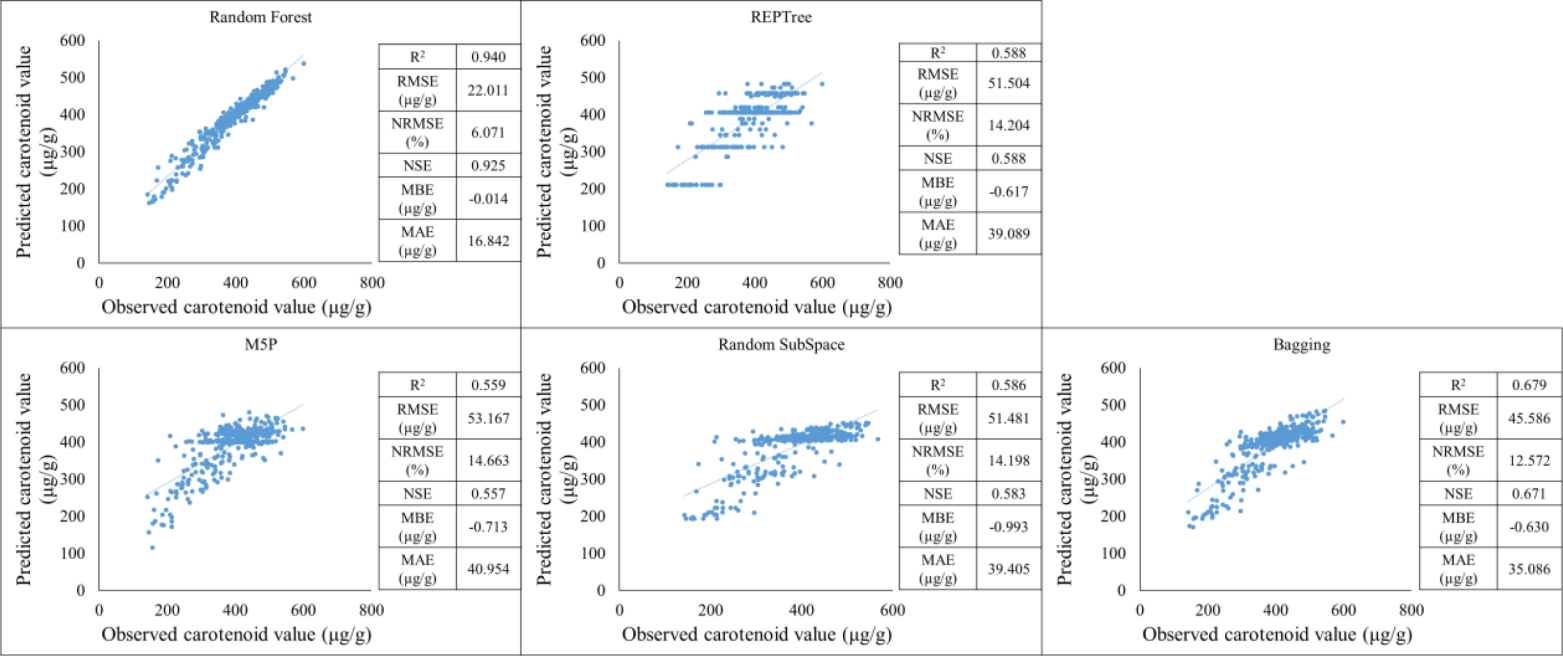
Machine learning results in establishing carotenoid prediction during the training period.

In the training period, it was observed that the Random forest (R^2^ = 0.940) had RMSE = 22.011 µg/g. MAE = 16.842 µg/g. MBE=-0.014 µg/g. NRMSE = 6.071% and the NSE = 0.925 which shows an almost perfect prediction. An R^2^ value of 0.94 indicates that the model is explanatory and fits the data very strongly. The RMSE and MAE values are moderately low. suggesting that the model performs well overall. The REPTree (R^2^ = 0.588) had RMSE = 51.504 µg/g. MAE = 39.089 µg/g. MBE= -0.617 µg/g. NRMSE = 14.204% and the NSE = 0.588 which shows a moderately strong predicton. The M5P (R^2^ = 0.559) had RMSE = 53.167µg/g. MAE = 40.954 µg/g. MBE= -0.713 µg/g. NRMSE = 14.663% and the NSE = 0.557 which shows a moderately strong predicton. The Random SubSpace (R^2^ = 0.586) had RMSE = 51.481 µg/g. MAE = 39.405 µg/g. MBE= -0.993 µg/g. NRMSE = 14.198% and the NSE = 0.583 which shows a moderately strong prediction. The Bagging (R^2^ = 0.679) had RMSE = 45.586 µg/g. MAE = 35.086 µg/g. MBE= -0.630 µg/g. NRMSE = 12.572% and the NSE = 0.671 which shows a moderately strong predicton (**Figure 15**).

**Figure 16:**
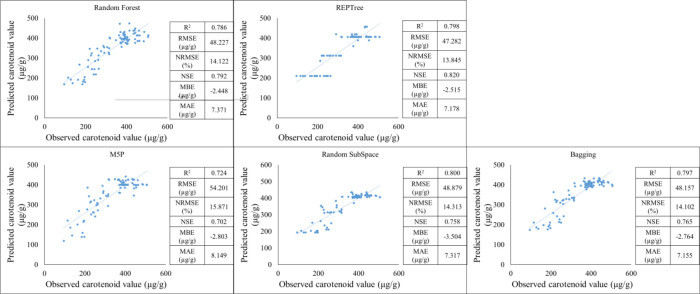
Machine learning results in establishing carotenoid prediction during testing period.

In the testing period, it was observed that the Random forest (R^2^ = 0.786) had RMSE = 48.227 µg/g. MAE = 7.371 µg/g. MBE= -2.448 µg/g. NRMSE = 14.122% and the NSE = 0.792, which shows an almost strong prediction. The REPTree (R^2^ = 0.798) had RMSE = 47.282 µg/g. MAE = 7.178µg/g. MBE= -2.515µg/g. NRMSE = 13.845% and the NSE = 0.820 which shows a strong prediction. The M5P (R^2^ = 0.724) had RMSE = 54.201 µg/g. MAE = 8.149 µg/g. MBE= -2.803 µg/g. NRMSE = 15.871% and the NSE = 0.702 which shows a strong predicton. The Random SubSpace (R^2^ = 0.800) had RMSE = 48.879 µg/g. MAE = 7.317 µg/g. MBE= -3.504 µg/g. NRMSE = 14.313% and the NSE = 0.758 which shows a strong predicton. The Bagging (R^2^ = 0.797) had RMSE = 48.157 µg/g. MAE = 7.155 µg/g. MBE= -2.764 µg/g. NRMSE = 14.102% and the NSE = 0.765 which shows a moderately strong predicton. In the testing period. the REPTree model gave the best results in the test period because of its high R² value of 0.798. which shows that the model explains the variability of the data well. and its low RMSE of 47.282 µg/g. which is the lowest error value compared to the other models. Low MAE = 7.178 µg/g which is one of the lowest absolute error values. MBE= -2.515 µg/g is an acceptable bias. Low NRMSE = 13.845% better relative performance than most models. The highest NSE = 0.820, which shows the best prediction performance on the test data. Although both Random Forest and Random SubSpace gave good results. In contrast, REPTree provided the most reliable and balanced performance during testing (R² = 0.798, NSE = 0.820), which we highlighted as the most suitable model for practical application (**Figure 16**). Conversely, REPTree and Random Forest showed more balanced prediction accuracy, highlighting the importance of proper parameter control and validation.

## 4 Discussion

This study investigated the potential of spectral data and machine learning for estimating carotenoid content in maize under different environmental conditions in the Pannonian region. Leaf carotenoid content showed a strong positive correlation with chlorophyll content (R² = 0.925), reflecting their coordinated regulation under non-stress conditions. While chlorophyll primarily governs photosynthetic capacity, carotenoids contribute both to light harvesting and photoprotection, emphasizing their importance in plant stress response and pigment balance. The high correlation observed here supports the use of carotenoid estimation as a complementary measure to chlorophyll assessment.

Traditional indices are not very sensitive to changes in carotenoid concentration, as their signals are mainly linked to the dominant absorption of chlorophyll and the near-infrared contrast determined by the leaf structure. In corn, this problem is further exacerbated by dense, vertically structured foliage, where multiple scattering and shading between leaf layers obscure pigment-specific spectral information. In addition, carotenoid content often varies non-linearly with canopy cover or leaf area index, so biomass-oriented indices are unable to distinguish subtle physiological changes in pigment ratios. These limitations justify the use of narrow-band, hyperspectral data-based, pigment-specific indices for carotenoid estimation, which are based directly on carotenoid absorption characteristics and spectral separation from chlorophyll. PRI was calculated from laboratory and field spectral measurements, specifically from plant-level spectral reflectance. This was typically a measurement from a hyperspectral spectrometer or narrowband radiometer. The PSSR was developed from leaf and canopy-level hyperspectral data. RARS-like indices also derive from hyperspectral spectral data, where different narrow wavelength bands were compared to pigment-sensitive signals. Carter’s carotenoid indices and modifications (e.g., mCRI) were also developed from hyperspectral or narrowband spectral reflectance for pigment-specific studies. This typically involved ground or aerial photography/spectroradiometric measurements (Gamon et al., 1990; Gitelson et al., 2002; Gitelson et al., 2003; Gitelson et al., 2006; Rodriguez-galiano et al., 2014; You et al., 2017; Zhou and Li, 2019; Smith, 2020; Bagherzadeh et al., 2021; Guo et al., 2021).

The method used in the present study is based directly on leaf-level spectral measurements. In the case of satellite or high-altitude aerial imaging, the spectral information of a single pixel is not determined solely by plant biomass, but also by the ratio of vegetation to soil, the optical properties of the soil, and the contribution of undergrowth and shaded surfaces. This spectral mixing is particularly significant in areas with heterogeneous land cover or in undeveloped stands, where soil reflection can play a dominant role. An additional source of uncertainty is the structure of the plant stand, including leaf angle, canopy layering, and spatial variability of leaf area index, all of which influence the absorption and scattering of incoming radiation. In addition, different plant species have different spectral reflectance characteristics due to their anatomical and biochemical properties, so in the case of species mixing, the spectral signals associated with individual pigments may be further distorted. In contrast, leaf-level measurements eliminate spectral mixing resulting from surface heterogeneity and structural differences, allowing for a more direct and accurate estimation of pigment content and physiological status.

Spectral reflectance analysis revealed that carotenoid concentration significantly affects the visible light spectrum, particularly between 500–650 nm, consistent with absorption features of lutein and β-carotene. The “red edge” region (680–700 nm) and NIR reflectance primarily reflected structural features of leaves rather than pigment concentration. These findings confirm that targeted hyperspectral measurements provide robust, non-destructive estimation of carotenoid content, aligning with previous studies using narrowband spectral measurements (Gitelson and Solovchenko (Afonso et al., 2017); Huang et al., 2018 (Koirala et al., 2020)).

Testing of existing carotenoid indices demonstrated poor predictive performance for the studied maize samples, with low R² and mostly negative NSE values. This underscores the need for customized spectral indices under specific crop and environmental conditions. Using principal component analysis, nine new indices (CAR_1_–CAR_9_) were developed, with CAR_7_, CAR_8_, and CAR_9_ showing the highest predictive accuracy across multiple training and testing periods. CAR_8_ consistently achieved the lowest RMSE, NRMSE, and MAE, while CAR_7_ performed best in terms of NSE, indicating excellent explanatory and predictive power. These results support the use of these indices as reliable tools for carotenoid estimation in maize.

Machine learning approaches further enhanced model performance. Among the five algorithms tested (Random Forest, REPTree, M5P, Random SubSpace, and Bagging), REPTree provided the most reliable and balanced predictions during testing (R² = 0.798, NSE = 0.820, RMSE = 47.282 µg/g), outperforming other methods in overall accuracy and bias control. Random Forest and Random SubSpace also performed well, demonstrating that advanced regression techniques can significantly improve prediction accuracy, consistent with previous studies in other crops (Afonso et al., 2017 (Zhang and Xue, 2024); Koirala et al., 2020 (Prilianti et al., 2021)).

The results of this study align with prior reports demonstrating the effectiveness of hyperspectral and machine learning approaches for pigment estimation. Zhang and Xue 2024 (Zhang and Xue, 2024) applied wet-lab extraction and nonlinear SVR models for carotenoid estimation in poplar leaves, achieving R² values comparable to those observed for CAR_7_ and CAR_8_ in this study. Similarly, Prilianti et al., 2021 (Prilianti et al., 2021) emphasized the potential of multispectral imagery and convolutional neural networks for non-destructive pigment estimation. These findings collectively demonstrate that both spectral indices and advanced statistical or machine learning techniques are essential for accurate, rapid, and non-invasive estimation of carotenoids in crops.

The integration of newly developed spectral indices with machine learning algorithms allows for robust, non-destructive estimation of maize carotenoid content. The use of active sensor technology under standardized lighting conditions ensured reproducible measurements and minimized environmental interference. Nevertheless, potential limitations exist when scaling to passive sensors or field applications under variable lighting, highlighting the need for further sensitivity analyses and calibration under open-field conditions. Furthermore, reliance on hyperspectral equipment may constrain practical application in resource-limited regions. Despite these challenges, CAR_7_, CAR_8_, and REPTree emerge as promising tools for high-accuracy carotenoid monitoring, supporting their future use in precision agriculture and stress assessment.

## 5 Conclusion

The results of this study clearly confirm the preliminary hypothesis that the use of spectral indices specifically optimized for corn significantly improves the accuracy of non-destructive estimation of carotenoid content compared to conventional general indices. The studies confirmed that carotenoids play a key role in the physiology of corn plants: not only contribute to photosynthesis as supplementary light-absorbing pigments, but also play a fundamental role in protecting against photooxidative stress. The close relationship between carotenoid and chlorophyll content confirms that the dynamics of these pigments sensitively reflect the current physiological state of the plant, especially under stress conditions.

The poor performance of traditional carotenoid indices has highlighted that they were developed for significantly different measurement environments and sensors (e.g., multispectral, wide-bandwidth systems used in satellite imagery or UAV platforms), where atmospheric effects, mixed pixels, and canopy and soil effects cause significant uncertainty. In contrast, the high spectral resolution leaf-level hyperspectral measurements used in the present study allowed for the accurate identification of wavelengths sensitive to carotenoids. The new corn-specific indices developed on this basis - especially CAR_7_, CAR_8_, and CAR_9_ - consistently showed better statistical performance in different years and under different environmental conditions, which clearly supports their validity.

The inclusion of machine learning methods further increased the reliability of the estimates, with the REPTree model proving to be the most balanced and stable solution in the testing phase. Based on the results presented, accurate, rapid, and non-destructive estimation of carotenoid content could become a realistic tool in precision agriculture, especially in the areas of stress monitoring and crop condition assessment. Future research could focus on scaling the new indices to the crop and remote sensing levels and validating them with UAV and satellite data, which could contribute to increasing the efficiency and sustainability of corn production in the long term.

## Data Availability

The original contributions presented in the study are included in the article/supplementary material. Further inquiries can be directed to the corresponding author.
